# Epigenetic Classification of Human Mesenchymal Stromal Cells

**DOI:** 10.1016/j.stemcr.2016.01.003

**Published:** 2016-02-09

**Authors:** Danilo Candido de Almeida, Marcelo R.P. Ferreira, Julia Franzen, Carola I. Weidner, Joana Frobel, Martin Zenke, Ivan G. Costa, Wolfgang Wagner

**Affiliations:** 1 Division of Stem Cell Biology and Cellular Engineering, Helmholtz-Institute for Biomedical Engineering, RWTH Aachen University Medical School, Pauwelsstraße 20, 52074 Aachen, Germany; 2 Department of Cell Biology, Institute for Biomedical Engineering, RWTH Aachen University Medical School, 52074 Aachen, Germany; 3 Department of Immunology, Institute of Biomedical Sciences, University of São Paulo, São Paulo 05508-000, Brazil; 4 Department of Cell Biology, IZKF Research Group Bioinformatics, Institute for Biomedical Engineering, RWTH Aachen University Medical School, 52074 Aachen, Germany; 5 Department of Statistics, Centre for Natural and Exact Sciences, Federal University of Paraiba, João Pessoa 58051-900, Brazil; 6 Helmholtz Institute for Biomedical Engineering, RWTH Aachen University, 52074 Aachen, Germany

**Keywords:** epigenetic score, mesenchymal stromal cell, mesenchymal stem cell, fibroblast, bone marrow, adipose tissue, DNA methylation

## Abstract

Standardization of mesenchymal stromal cells (MSCs) is hampered by the lack of a precise definition for these cell preparations; for example, there are no molecular markers to discern MSCs and fibroblasts. In this study, we followed the hypothesis that specific DNA methylation (DNAm) patterns can assist classification of MSCs. We utilized 190 DNAm profiles to address the impact of tissue of origin, donor age, replicative senescence, and serum supplements on the epigenetic makeup. Based on this, we elaborated a simple epigenetic signature based on two CpG sites to classify MSCs and fibroblasts, referred to as the Epi-MSC-Score. Another two-CpG signature can distinguish between MSCs from bone marrow and adipose tissue, referred to as the Epi-Tissue-Score. These assays were validated by site-specific pyrosequencing analysis in 34 primary cell preparations. Furthermore, even individual subclones of MSCs were correctly classified by our epigenetic signatures. In summary, we propose an alternative concept to use DNAm patterns for molecular definition of cell preparations, and our epigenetic scores facilitate robust and cost-effective quality control of MSC cultures.

## Highlights

• Mesenchymal stromal cells and fibroblasts have different DNA methylation patterns
• A two-CpG signature can distinguish between MSCs and fibroblasts
• Another two-CpG signature can discern MSCs from bone marrow and adipose tissue
• Subclones of MSCs are correctly classified by these epigenetic scores

## Graphical Abstract

**Figure:**
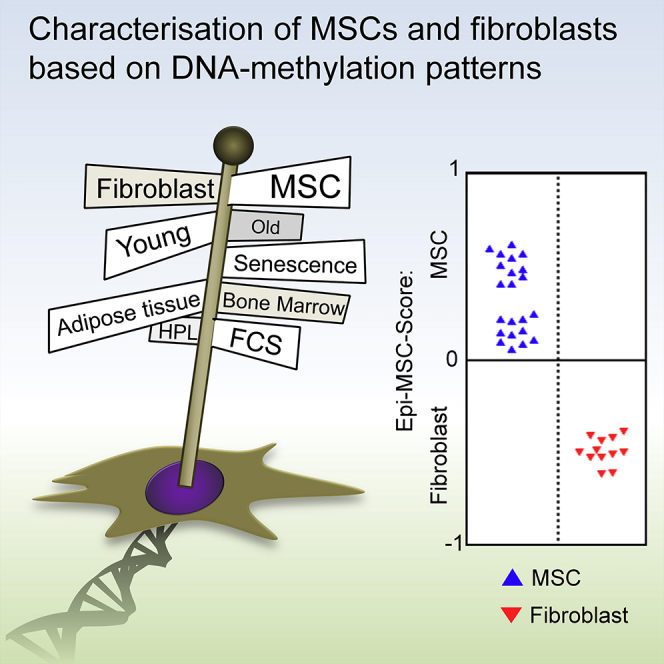

## Teaser

In this article, Wagner and colleagues show that specific DNA methylation patterns can be used for classification of mesenchymal stromal cells (MSCs). They provide simple epigenetic signatures based on two CpG dinucleotides to either discern MSCs and fibroblasts, or to demonstrate whether MSCs were isolated from adipose tissue or bone marrow. These assays can support quality control of cell preparations.

## Introduction

Mesenchymal stromal cells (MSCs) are currently tested for a wide range of clinical applications (Squillaro et al., 2015), but there are no precise measures for their quality control. Molecular markers to clearly discern MSCs and fibroblasts remain elusive. The major difference between these two cell types is that particularly MSCs comprise a multipotent subset often referred to as “mesenchymal stem cells” (Dominici et al., 2006). Several surface markers have been suggested for enrichment of MSCs, such as CD106, CD146, and CD271 (Buhring et al., 2007, Halfon et al., 2011, Sorrentino et al., 2008), but none of them seems to be exclusively expressed on MSCs. Proteomics and gene-expression profiles can discern cells that have been obtained from different tissues or under different culture conditions (Holley et al., 2015, Ishii et al., 2005), and high-content screening assays based on microRNA or RNAi can elucidate cell type-specific responses (Bae et al., 2009, Erdmann et al., 2015). However, all these profiling and high-throughput techniques are relatively time and labor consuming, require complex computational analysis, and can hardly be standardized for quality control of MSC preparations.

Cellular differentiation is reflected by specific epigenetic patterns. DNA methylation (DNAm) is the best characterized epigenetic modification, where cytosine guanine dinucleotides (CpGs) are covalently methylated at the cytosine residue (Jaenisch and Bird, 2003). DNAm has several advantages as a biomarker for classification of cell preparations: (1) it is rather stable; (2) it facilitates quantitative analysis at single-nucleotide resolution, and (3) it is directly coupled to cellular differentiation (Karnik and Meissner, 2013). We have recently described that DNAm levels at two CpGs can reliably discern between pluripotent and non-pluripotent cells (Lenz et al., 2015). In this study, we followed the hypothesis that the DNAm profile of MSCs might also reflect specific modifications that are indicative for the cell type and/or the tissue of origin. Small epigenetic signatures based on site-specific analysis of DNAm in a few CpG sites might therefore be particularly appealing for the classification of MSCs.

## Results

### Global Comparison of DNA Methylation Profiles

We compiled a well-curated dataset of publicly available DNAm profiles that were generated on the Illumina HumanMethylation BeadChip platforms: 83 DNAm profiles analyzed on 27K BeadChips were used as a training set; and 107 DNAm profiles of 450K BeadChips were used as independent validation sets (Tables S1 and S2). Therefore, we focused on 25,014 CpGs that were represented by both platforms. Initially, we performed principal-component analysis (PCA) to estimate the impact of cell type (MSCs or fibroblasts), tissue source (bone marrow [BM], adipose tissue [AT], lung, dermis, etc.), age (stratified by 40 years), passage (stratified by P5), or serum supplement (human platelet lysate [hPL] versus fetal calf/bovine serum [FBS]) on the global DNAm patterns. However, none of the major PCA components could clearly classify cell preparations according to these parameters, and there was only a moderate tendency in the comparisons: MSCs versus fibroblasts, and MSCs derived from BM versus AT (Figure S1).

**Figure 1:**
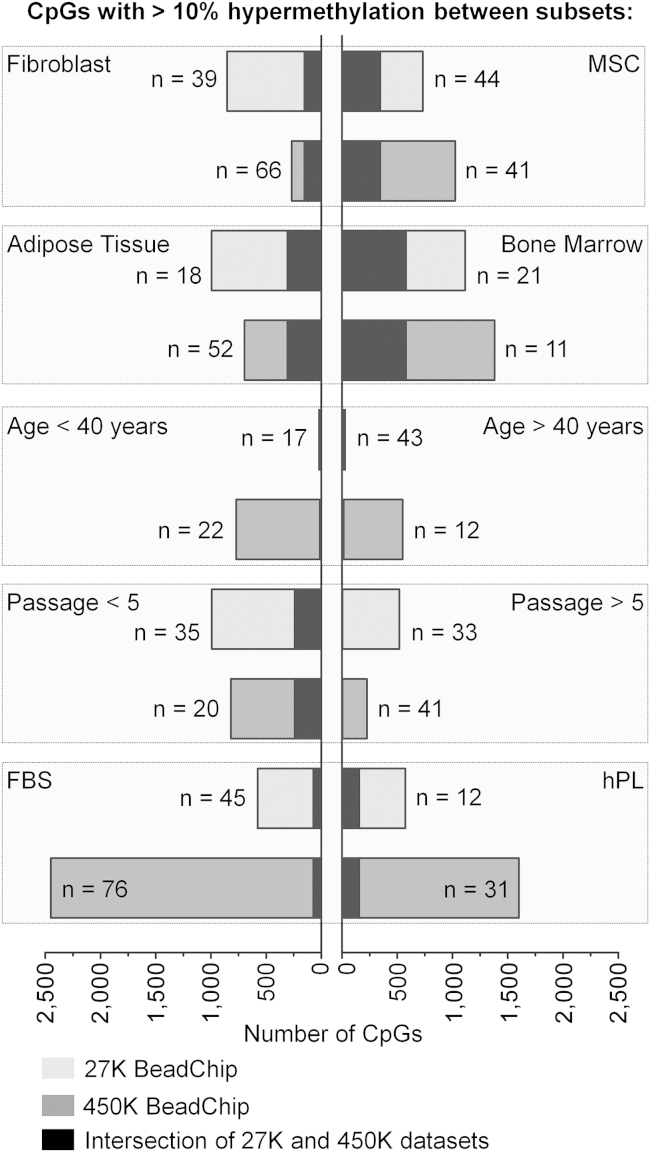
Differentially Methylated CpGs in Pairwise Comparisons DNA methylation profiles (generated on Illumina HumanMethylation BeadChips 27K or 450K) were stratified by cell type (MSCs versus fibroblasts), tissue source (here particularly MSCs from bone marrow versus adipose tissue), passage (<P5 or >P5), age (<40 or >40 years), and serum supplements in culture media (human platelet lysate [hPL] versus fetal calf/bovine serum [FBS]). The number of DNAm profiles per group is indicated (n) as well as the number of significant CpGs (adjusted limma t test: p < 0.05 and >10% difference in mean DNAm). Overlapping CpGs in the 27K and 450K datasets are indicated by black bars.

Subsequently, we determined the number of differentially methylated CpGs in pairwise comparisons (adjusted limma t test: p < 0.05 and at least 10% differential DNAm level). This was performed independently for the 27K-BeadChip training and the 450K-BeadChip validation set. To roughly estimate the reproducibility of DNAm differences, we then focused on CpGs with overlapping DNAm changes in both datasets (Figure 1): 346 and 152 CpGs were methylated higher in MSCs and fibroblasts, respectively, indicating that there are reproducible epigenetic differences between the two cell types. Furthermore, 580 and 307 CpGs were differentially methylated in MSCs from BM versus AT. There were hardly any overlapping age-related DNAm differences in samples from younger or older donors, although it has been shown that age-related DNAm patterns persist in MSCs (Frobel et al., 2014, Weidner et al., 2014). This might be due to the classification into two age groups, whereas age-related changes are continuously acquired throughout life. In analogy, we observed only 242 CpGs that were methylated higher at early passages (<P5) compared with late passages (>P5), although many DNAm changes were shown to be continuously hyper- and hypomethylated during culture expansion (Koch et al., 2013). Serum supplements seemed to induce rather few DNAm changes. Taken together, global analysis indicated that particularly cell type and tissue of origin are reflected by specific DNAm changes.

### Epigenetic Score for Classification into MSCs and Fibroblasts

**Figure 2:**
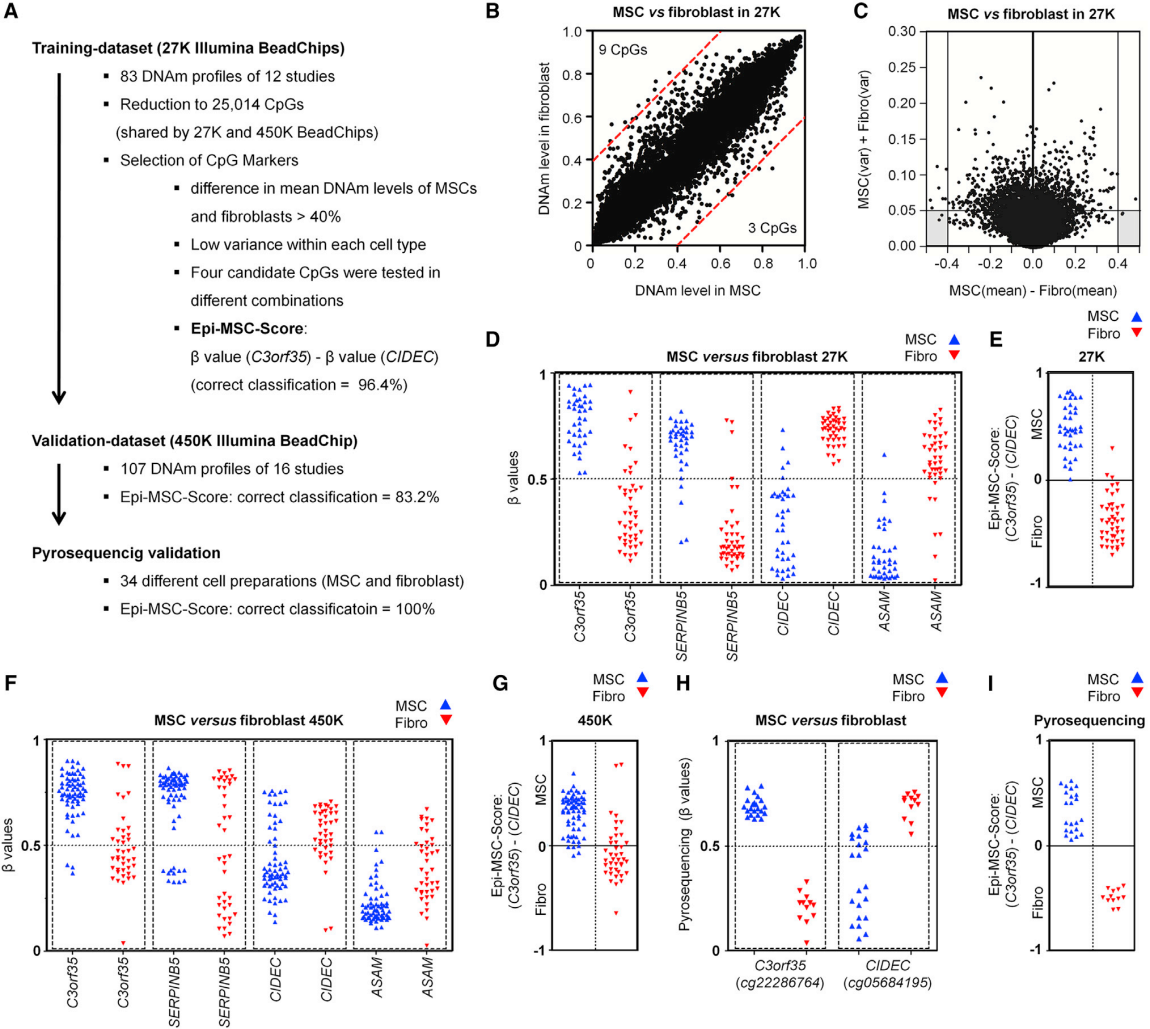
Epigenetic Classification of MSCs and Fibroblasts (A) Schematic overview of the experimental design that led to the Epi-MSC-Score. (B) Scatterplot of mean DNAm levels of MSCs and fibroblasts in the training dataset (CpGs with more than 40% difference are indicated by red lines). (C) Differential DNAm levels were plotted against the sum of variances within MSCs and fibroblasts. (D) DNAm levels (β values) of four CpGs that have been selected from the training datasets (27K BeadChips). (E) Classification of the training dataset by the Epi-MSC-Score. This score represents the difference of β values at cg22286764 (*C3orf35*) and cg05684195 (*CIDEC*). (F) DNAm levels of the four selected CpGs in the validation dataset (450K BeadChips; in analogy to Figure 2D). (G) Classification of the validation dataset by the Epi-MSC-Score. (H) Pyrosequencing analysis of DNAm at the two CpGs corresponding to the Epi-MSC-Score in 34 different cell preparations. (I) Classification of pyrosequencing results by the Epi-MSC-Score based on CpG in *C3orf35* and *CIDEC* as indicated.

To identify CpGs that facilitate the best discrimination of MSCs and fibroblasts in the 27K-BeadChip training set, we selected CpGs with (1) the highest difference in mean DNAm in MSCs versus fibroblasts, and (2) small variation in DNAm levels within each of the two cell types (Figure 2A). Only three and nine CpGs revealed more than 40% higher DNAm levels in MSCs and fibroblasts, respectively (Figure 2B). These CpGs were subsequently plotted against the sum of variances in MSCs and fibroblasts, and thereby we identified four candidate CpGs that were associated with serpin peptidase inhibitor B5 (*SERPINB5*: cg00226904), chromosome 3 open reading frame 35 (*C3orf35*: cg22286764), cell death-inducing DFFA-like effector C (*CIDEC*: cg05684195), and adipocyte-specific adhesion molecule (*ASAM*: cg19096475; Figures 2C and 2D). Iterative pair combinations of these CpGs demonstrated that the difference in DNAm at the CpGs in *C3orf35* and *CIDEC*, subsequently referred to as the Epi-MSC-Score, could best discern MSCs from fibroblasts: a positive score is indicative of MSCs and 96% of samples were correctly classified in the 27K-BeadChip training set (Figure 2E). We repeated the analysis after resampling the training set with bootstrapping, and the two CpGs were among the top eight stable CpG sites (Supplemental Experimental Procedures). In the independent 450K-BeadChip validation set, all four candidate CpGs revealed the same trend (Figure 2F) and 83% of the samples were classified correctly (Figure 2G). Overall the differences in mean DNAm levels in MSCs versus fibroblasts were smaller in this dataset. However, applying the two aforementioned criteria for selection of relevant CpGs on the 450K dataset demonstrated that the two CpGs in *C3orf35* and *CIDEC* were again among the best performing (data not shown).

We then designed pyrosequencing assays for these two regions to facilitate robust and more quantitative analysis of the DNAm levels at the two relevant CpG sites (Figure S2A). These pyrosequencing assays were tested on 34 primary cell preparations, all of which were correctly classified into MSCs and fibroblasts (Figures 2H and 2I). Gene-expression profiles demonstrated slightly higher expression of *C3orf35* and *CIDEC* in MSCs (Figure S2B). Thus, the Epi-MSC-Score can be used for the classification of MSCs and fibroblasts.

### Epigenetic Score to Discern MSCs from Bone Marrow and Adipose Tissue

**Figure 3:**
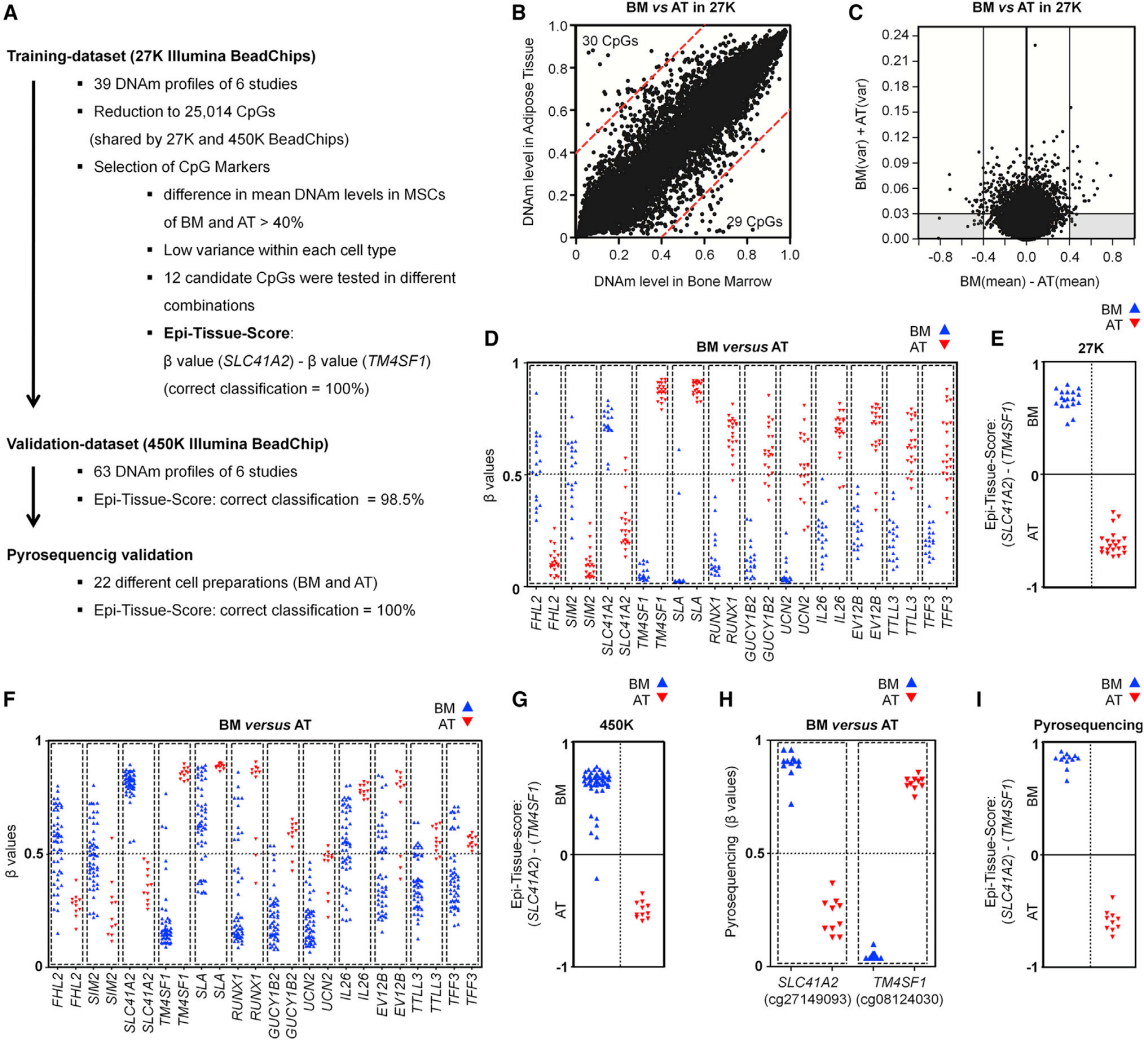
Classification of MSCs from Bone Marrow and Adipose Tissue (A) Schematic overview of experimental design that led to the Epi-Tissue-Score. (B) Scatterplot of mean DNAm levels in MSCs from bone marrow (BM) versus MSCs from adipose tissue (AT) in the training set (27K BeadChips; CpGs with more than 40% difference are indicated by red lines). (C) Differential DNAm levels were plotted against the sum of variances within MSCs derived from either BM or AT. (D) β Values (DNAm levels) of 12 CpGs that were selected by these criteria. (E) Classification of the training dataset by the Epi-Tissue-Score. This score represents the difference of β values at cg27149093 (*SLC41A2*) and cg08124030 (*TM4SF1*). (F) DNAm levels of the 12 selected CpGs in the validation dataset (450K BeadChips; in analogy to Figure 3D). (G) Classification of the validation dataset by the Epi-Tissue-Score. (H) Pyrosequencing analysis of DNAm at the two CpGs corresponding to the Epi-Tissue-Score in 22 MSC samples from BM and AT. (I) Classification of pyrosequencing results by the Epi-Tissue-Score based on CpG in *SLC41A2* and *TM4SF1* as indicated.

We extended this analysis to derive an “Epi-Tissue-Score” for discerning MSCs that were initially isolated from either BM or AT, since these tissues are most frequently used for isolation of MSCs (Figure 3A). 29 and 30 CpGs revealed a more than 40% higher mean DNAm level in MSCs from either BM or AT, respectively (Figure 3B). We focused on 12 CpGs with lowest variances within each of these groups, which were associated with: solute carrier family 41 magnesium transporter member 2 (*SLC41A2*: cg27149093); single-minded family BHLH transcription factor 2 (*SIM2*: cg02672220); four and a half LIM domains 2 (*FHL2*: cg10635061); transmembrane 4 six family member 1 (*TM4SF1*: cg08124030); src-like-adaptor (*SLA*: cg02794695); runt-related transcription factor 1 (*RUNX1*: cg19836199); guanylate cyclase 1, soluble, beta 2 (*GUCY1B2*: cg16692277); urocortin 2 (*UCN2*: cg05125838); interleukin-26 (*IL26*: cg25697314); ecotropic viral integration site 2B (*EVI2B*: cg05109049); tubulin tyrosine ligase-like family member 3 (*TTLL3*: cg03375833); and intestinal trefoil factor 3 (*TFF3*: cg04806409; Figures 3C and 3D). The difference between the DNAm levels of the CpGs in *SLC41A2* and *TM4SF1* showed best discrimination in the 27K-BeadChip training set (100% correctly classified) and was therefore considered as the Epi-Tissue-Score (Figure 3E). Notably, all 12 candidate CpGs demonstrated tissue type-specific DNAm patterns also in the 450K-BeadChip validation set (Figure 3F), and 98.4% of these samples were correctly classified by the Epi-Tissue-Score (Figure 3G). Pyrosequencing assays were designed for the two CpGs in *SLC41A2* and *TM4SF1* (Figure S3A), and thereby 22 analyzed MSC preparations were correctly classified into BM- or AT-derived MSCs (Figures 3H and 3I). We also observed moderate differences in gene expression of *SLC41A2* and *TM4SF1* between MSCs from BM and AT (Figure S3B). Our analysis pinpoints clear molecular differences in MSCs that have been isolated from BM or AT, which can be reliably tracked by the Epi-Tissue-Score.

### Epigenetic Classification of iPSC-Derived MSCs

We have recently demonstrated differentiation of induced pluripotent stem cells (iPSCs) toward MSCs, referred to as iPS-MSCs (Frobel et al., 2014). The DNAm profiles of these iPS-MSCs were now compared with those of primary cell preparations: iPS-MSCs were classified as MSCs by the Epi-MSC-Score (Figures S4A and S4B), and this was validated by pyrosequencing analysis of additional iPS-MSC preparations (Figure S4F). In contrast, the DNAm patterns at the 12 tissue-specific CpGs were not clearly indicative of BM- or AT-derived MSCs (Figure S4C). PCA analysis using either the four cell type-specific or the 12 tissue-specific CpGs supported the notion that iPS-MSCs are related to MSCs, whereas they do not reflect a clear tissue-specific association (Figures S4D and S4E). This is in line with our previous report that tissue-specific patterns are erased by reprogramming into iPSCs (Shao et al., 2013), and overall are not reestablished upon differentiation of iPSCs toward MSCs (Frobel et al., 2014).

### Epigenetic Classification of Subclones

**Figure 4:**
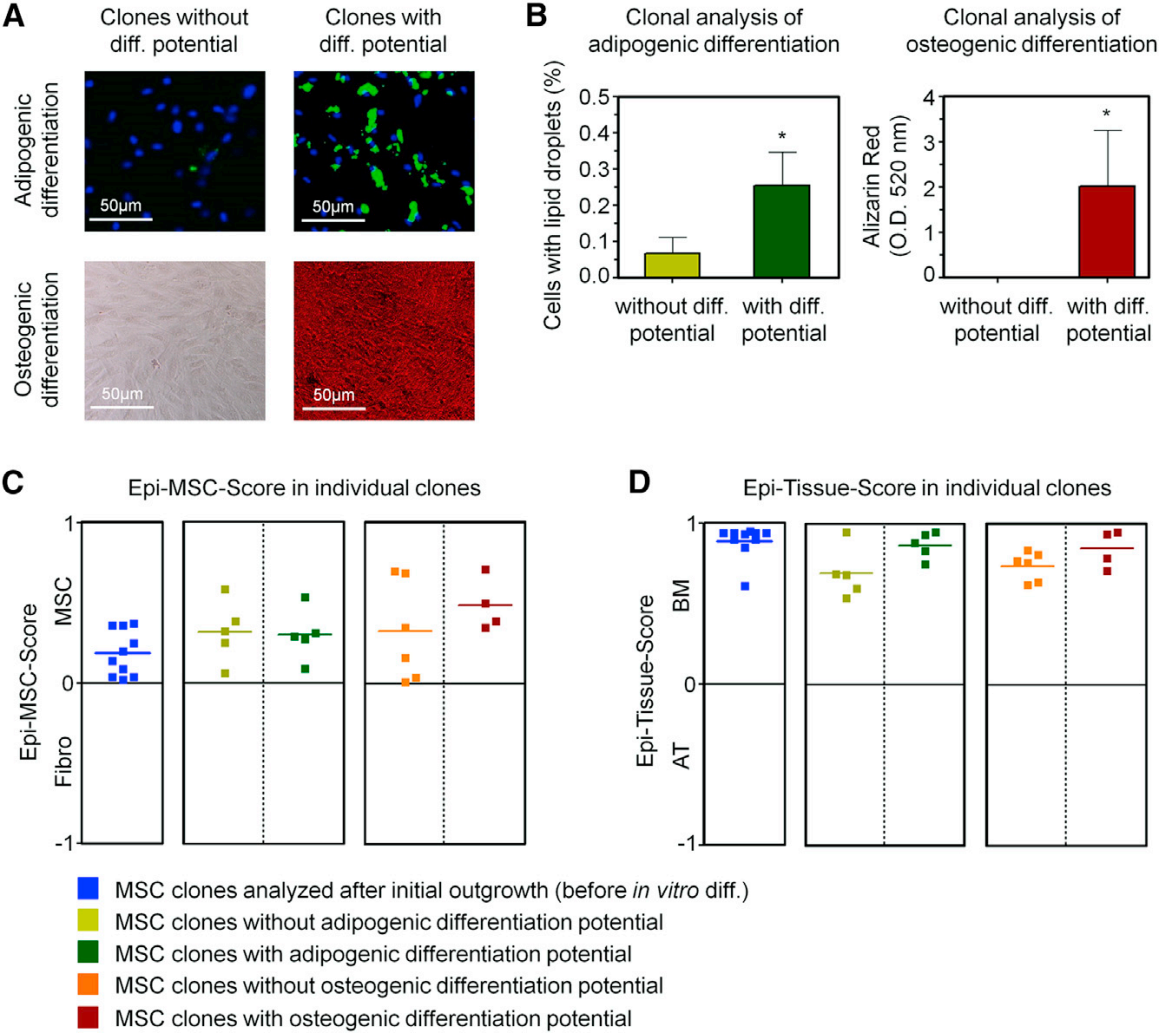
Analysis of Epigenetic Scores in Subclones of MSCs (A) Bone marrow-derived MSCs were subcloned and differentiated toward adipogenic or osteogenic lineages (stained with BODIPY/DAPI or Alizarin red, respectively). Representative images of clones with low or high differentiation potential are shown. (B) The in vitro differentiation potential toward adipogenic and osteogenic lineages was determined based on the percentage of cells with fat droplets or absorbance of Alizarin staining, respectively. For subsequent pyrosequencing analysis, we selected five clones that revealed either higher or lower differentiation (Student's t test; ^∗^p < 0.05; error bars represent the SD). (C and D) Classification of MSC clones based on pyrosequencing results by Epi-MSC-Score (C) and Epi-Tissue-Score (D).

Mesenchymal stem cells comprise heterogeneous subpopulations (Cai et al., 2014, Schellenberg et al., 2012), and we have therefore challenged our epigenetic signatures on subclones. MSC cultures were seeded in 96-well plates in limiting dilutions and analyzed after 2 weeks. Additional 96-well plates were further differentiated toward adipogenic or osteogenic lineages for 2 weeks (Figure S4G). The individual subclones revealed very heterogeneous in vitro differentiation potential, as described in our previous work (Schellenberg et al., 2012), and could therefore be classified into clones with high or low differentiation potential (Figure 4A). Adipogenic differentiation potential was estimated by the percentage of cells harboring fat droplets (stained with BODIPY) and osteogenic differentiation by the amount of calcium phosphate precipitates (stained with Alizarin red; Figure 4B). DNA of 30 clones was subsequently harvested and analyzed with our Epi-MSC-Score and Epi-Tissue-Score. All subclones were correctly classified as BM-derived MSCs, irrespective of their in vitro differentiation potential (Figures 4C, 4D, S4H, and S4I). This indicates that the epigenetic classification is not due to shifts in the cellular composition, and rather reflects cell-intrinsic molecular characteristics.

## Discussion

Reliable measures for quality control are a prerequisite for the standardization of cell preparations to be used in experimental studies and cellular therapy. Here, we demonstrate that epigenetic signatures can support the classification of MSCs. In general, the precision of signatures can be increased by using a higher number of CpGs, but this requires more complex or even genome-wide analysis. Our two CpGs scores, which are based on one hypermethylated and one hypomethylated CpG site, are therefore a tradeoff to facilitate fast, cost-effective, and transparent classification.

Despite extensive efforts, it remains a challenge to distinguish between fibroblasts and MSCs. This definition is usually based on the in vitro differentiation potential of MSCs, although these surrogate assays hardly facilitate quantitative comparison, particularly not between different laboratories (Bortolotti et al., 2015, Dominici et al., 2006, Hematti, 2012). In our comparative study, we had to rely on the classification provided by the authors who deposited the DNAm profiles. Hence, they are not based on common standards in cell culture and quality control. At least for the cell preparations that we analyzed by pyrosequencing, we consistently observed higher differentiation potential in MSCs compared with fibroblasts (Koch et al., 2011), and these were all correctly classified by the Epi-MSC-Score. On the other hand, our clonal analysis indicated that this signature is not directly associated with the subset in MSCs that reveals higher in vitro differentiation potential.

The epigenome reflects the tissue of origin even after long-term culture (Reinisch et al., 2015, Schellenberg et al., 2012). MSCs can be isolated from a multitude of different tissues (Crisan et al., 2008), but the vast majority of studies utilize MSCs from BM and AT. In fact, cell preparations derived from other tissues are often rather referred to as fibroblasts, and therefore classification of the Epi-MSC-Score may partly be also attributed to the different tissue sources. Either way, classifications with the Epi-MSC-Score are generally in line with those provided by the corresponding publications. Furthermore, the Epi-Tissue-Score can very reliably distinguish between MSCs from BM and AT. The remarkable difference in the epigenetic makeup of MSCs from different tissues, which are cell intrinsic and not due to cellular heterogeneity, may reflect the stark tissue-specific differences in gene-expression profiles (Wagner et al., 2005), proteome (Wagner et al., 2006), and functional readouts (Reinisch et al., 2015). All the more, such analysis is relevant for quality control.

Researchers are usually aware of the tissue that was initially used for isolation of MSCs, but there is evidence that accidental interchange of samples or contaminations with other cells can occur (Garcia et al., 2010, Torsvik et al., 2010). For established cell lines, some contaminations can be detected by specific SNPs or mutations, but for primary cells with unknown genetic background this can hardly be unraveled. In this regard, our epigenetic signatures provide a perspective for quality control of cell preparations. We expect that the signatures can be further fine-tuned based on the rapidly growing number of available DNAm datasets. This will also facilitate generation of other epigenetic signatures reflecting functional properties of MSCs, such as their immunomodulatory potential or the hematopoiesis supportive function (Wuchter et al., 2015). It is even conceivable that epigenetic signatures can be developed to estimate the therapeutic potential of MSCs, but such predictors need to be specifically trained and validated on suitable datasets. In this regard, our exploratory study provides an alternative concept for the definition, characterization, and classification of MSCs.

## Experimental Procedures

A detailed description of all Experimental Procedures used is presented in Supplemental Experimental Procedures.

### DNA Methylation Datasets

Illumina Human Methylation BeadChip datasets (27K or 450K) of MSCs and fibroblasts were retrieved from the NCBI Gene Expression Omnibus (Tables S1 and S2).

### Derivation of Epigenetic Scores

To identify the best suited biomarkers for classification, we selected CpG sites with high differences in mean DNAm levels (>40% of difference) and low variance within groups. A hypermethylated and a hypomethylated CpG were then utilized for each score as follows: Epi-MSC-Score = β value at cg22286764 (*C3orf35*) minus the β value at cg05684195 (*CIDEC*); and Epi-Tissue-Score = β value at cg27149093 (*SLC41A2*) minus the β value at cg08124030 (*TM4SF1*). Both scores range from −1 to 1; positive values indicate MSCs and BM, and negative ones fibroblast and AT, respectively.

### Primary Cells

All cells were taken after written consent was granted, and have been specifically approved by the local Ethics Committees for Use of Human Subjects at RWTH Aachen University (permit numbers: BM-MSC: *#EK128/09*; AT-MSCs: *#EK187/08*; fibroblasts: *#EK187/08*). Cell culture, immunophenotyping, and in vitro differentiation were performed as described previously (Frobel et al., 2014, Koch et al., 2011). Additional Information about the samples is provided in Table S3. For clonal analysis, MSCs at passage 1–2 (n = 3) were submitted to the limiting dilutions in 96-well plates of 1, 3, 10, and 30 cells per well as described previously (Schellenberg et al., 2012).

### Pyrosequencing Analysis

Genomic DNA was isolated from 10^6^ cells (bulk culture) or clones in 96-well plates using the NucleoSpin Tissue/Tissue XS kits (Macherey-Nagel) and quantified with an ND-1000 spectrometer (NanoDrop). Between 100 and 1,000 ng of DNA was sodium bisulfite-converted using the EZ DNA Methylation kit (Zymo Research), and PCR procedures and sequencing assays were performed using the PyroMark PCR and Q96 kits (Qiagen) (Lenz et al., 2015). Primers are specified in Table S4.

## Supplemental Information

Document S1. Supplemental Experimental Procedures, Figures S1–S4, and Tables S1–S4

Document S2. Article plus Supplemental Information
