# Supplementary material for: Epigenetic Classification of Human Mesenchymal Stromal Cells
Source: Stem Cell Reports. 2016 Feb 9;6(2):168–75. doi: 10.1016/j.stemcr.2016.01.003 (PMC4750140; doi:10.1016/j.stemcr.2016.01.003)
Supplement: Document S1. Supplemental Experimental Procedures, Figures S1–S4, and Tables S1–S4 [file mmc1.pdf]

Stem Cell Reports, Volume *6*

Supplemental Information

Epigenetic Classification of Human Mesenchymal Stromal Cells

Danilo Candido de Almeida, Marcelo R.P. Ferreira, Julia Franzen, Carola I. Weidner, Joana Frobel, Martin Zenke, Ivan G. Costa, and Wolfgang Wagner

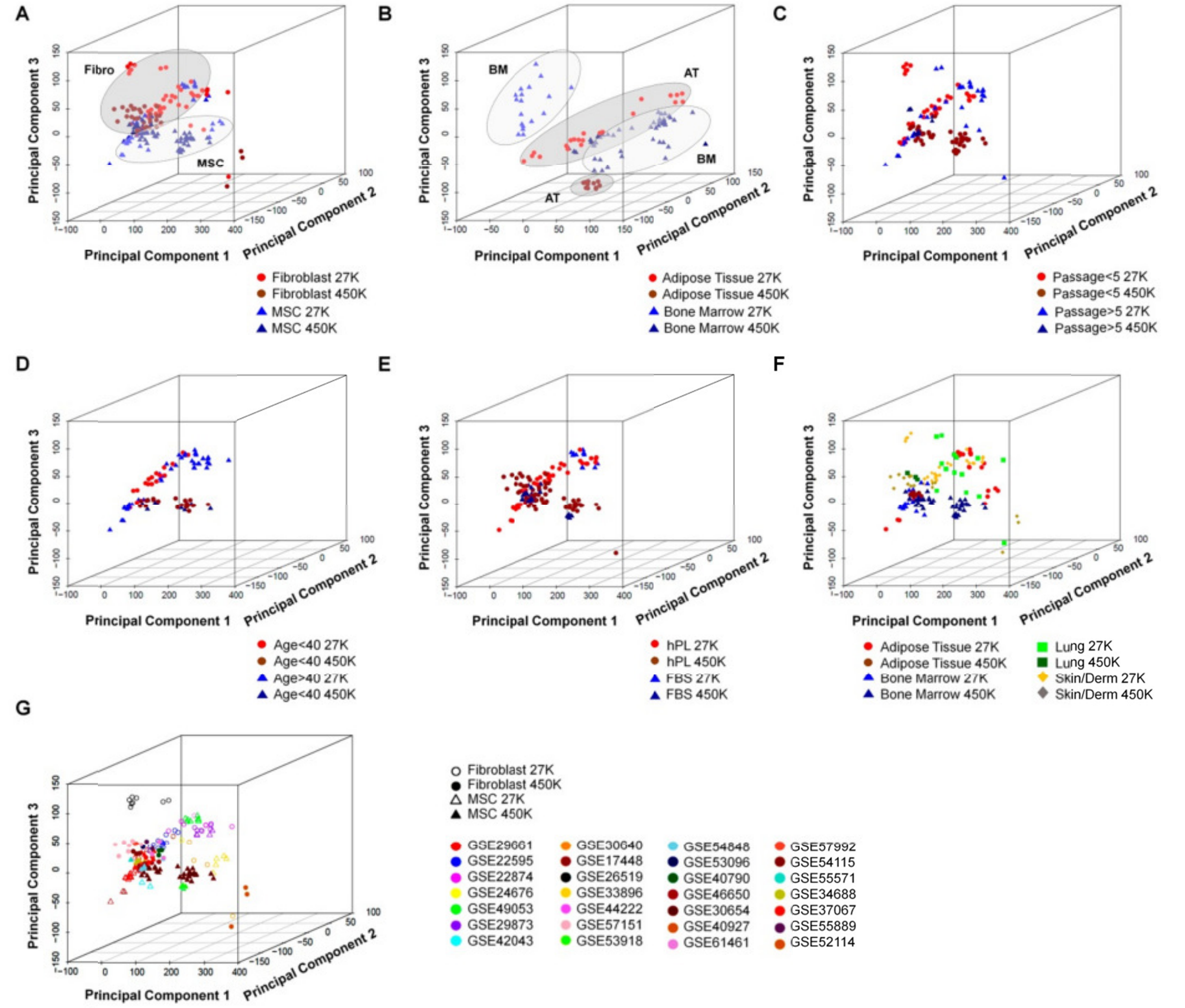

Figure S1. Principal component analysis of DNAm patterns, related to Figure 1.
Principal component analysis (PCA) of DNAm profiles according to:
(A) cell type (MSCs *versus* Fibroblasts),
(B) tissue source (MSCs from bone marrow [BM] or adipose tissue [AT]),
(C) passage (less or more than 5 passages),
(D) age (less or more than 40 years),
(E) serum supplement utilized in culture (human platelet lysate [hPL] or fetal bovine serum [FBS]), and
(F) different tissue sources,
(G) different studies (GEO IDs of the corresponding datasets are indicated)
Overall, none of these cellular characteristics was clearly reflected by major components in PCA. A moderate association was only observed for cell type and comparison of MSCs from BM and AT (indicated by shaded circular areas). Samples of individual studies clustered often together, but this may not only be due to technical reasons or normalization regimen, but rather to the different cell preparations and culture conditions used in these individual studies.

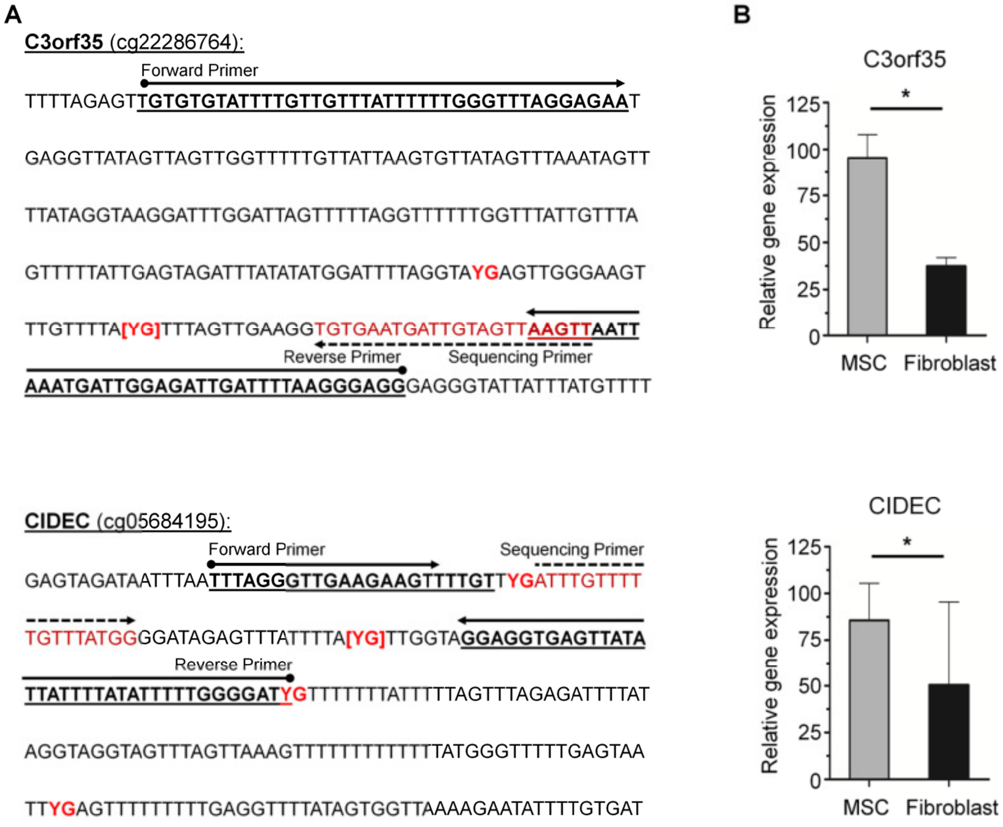

Figure S2. Pyrosequencing and gene expression for Epi-MSC-Score, related to Figure 2.
(A) Pyrosequencing assays for the two relevant CpG-sites in the Epi-MSC-Score. The genomic sequence of bisulfite-converted DNA is demonstrated. Please note that all non-methylated cytosines have been converted to thymidines; CG base pairs are therefore indicated as YGs. The relevant CpG-sites (cg22286764 and cg05684195) are highlighted in red brackets.
(B) Gene expression levels of *C3orf35* and *CIDEC* (associated with CpGs of the Epi-MSC-Score). The gene expression profiles were downloaded from GEO (all Illumina HumanHT-12 v4 platform; n = 46). Data were quantile normalized and relative gene expression levels are indicated. * $p < 0.05$ (Student's t-test).

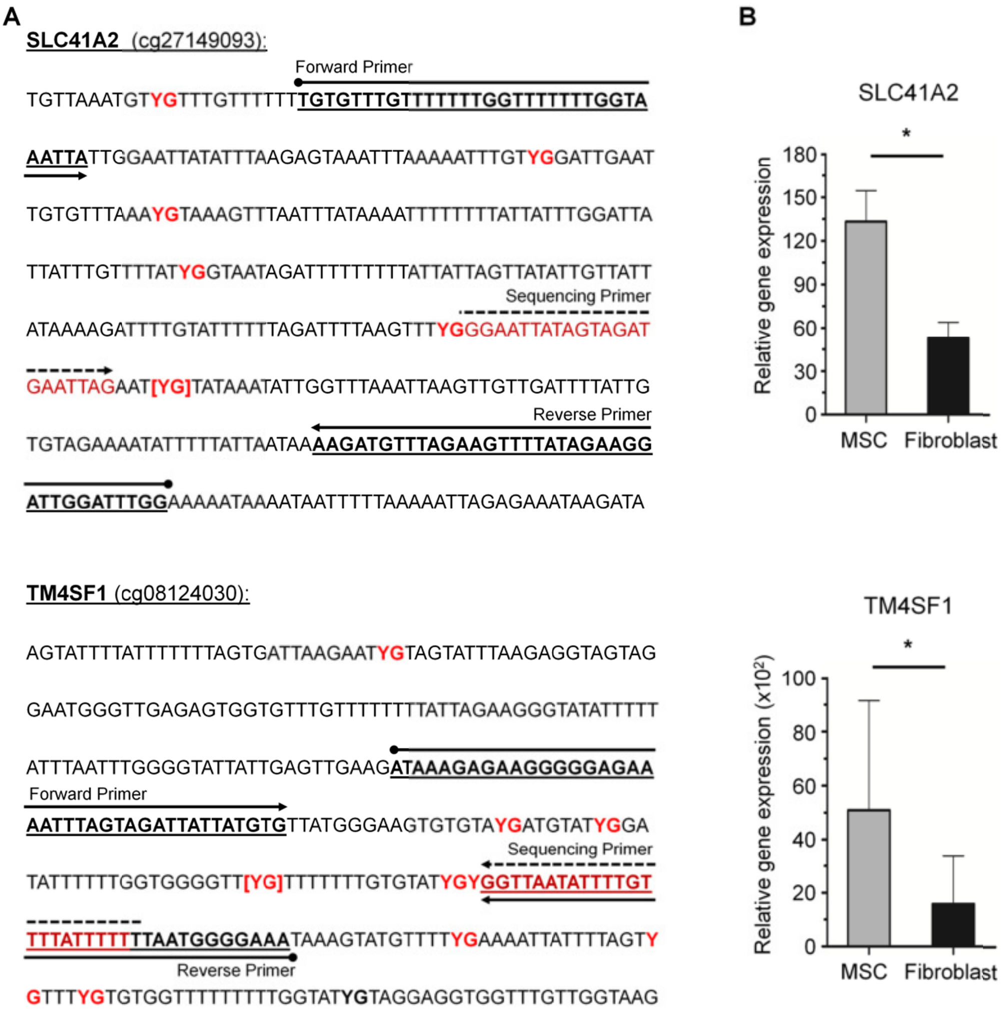

Figure S3. Pyrosequencing and gene expression for Epi-Tissue-Score, related to Figure 3.
(A) Pyrosequencing assays for the two relevant CpG-sites in the Epi-Tissue-Score. The genomic sequence of bisulfite-converted DNA is demonstrated. Please note that all non-methylated cytosines have been converted to thymidines; CG base pairs are therefore indicated as YGs. The relevant CpG-sites (cg27149093 and cg08124030) are highlighted in red brackets.
(B) Gene expression levels of *SLC41A2* and *TM4SF1* (associated with CpGs of the Epi-Tissue-Score). The gene expression profiles were downloaded from GEO (all Illumina HumanHT-12 v4 platform; n = 24). Data were quantile normalized and relative gene expression levels are indicated. * $p < 0.05$ (Student's t-test).

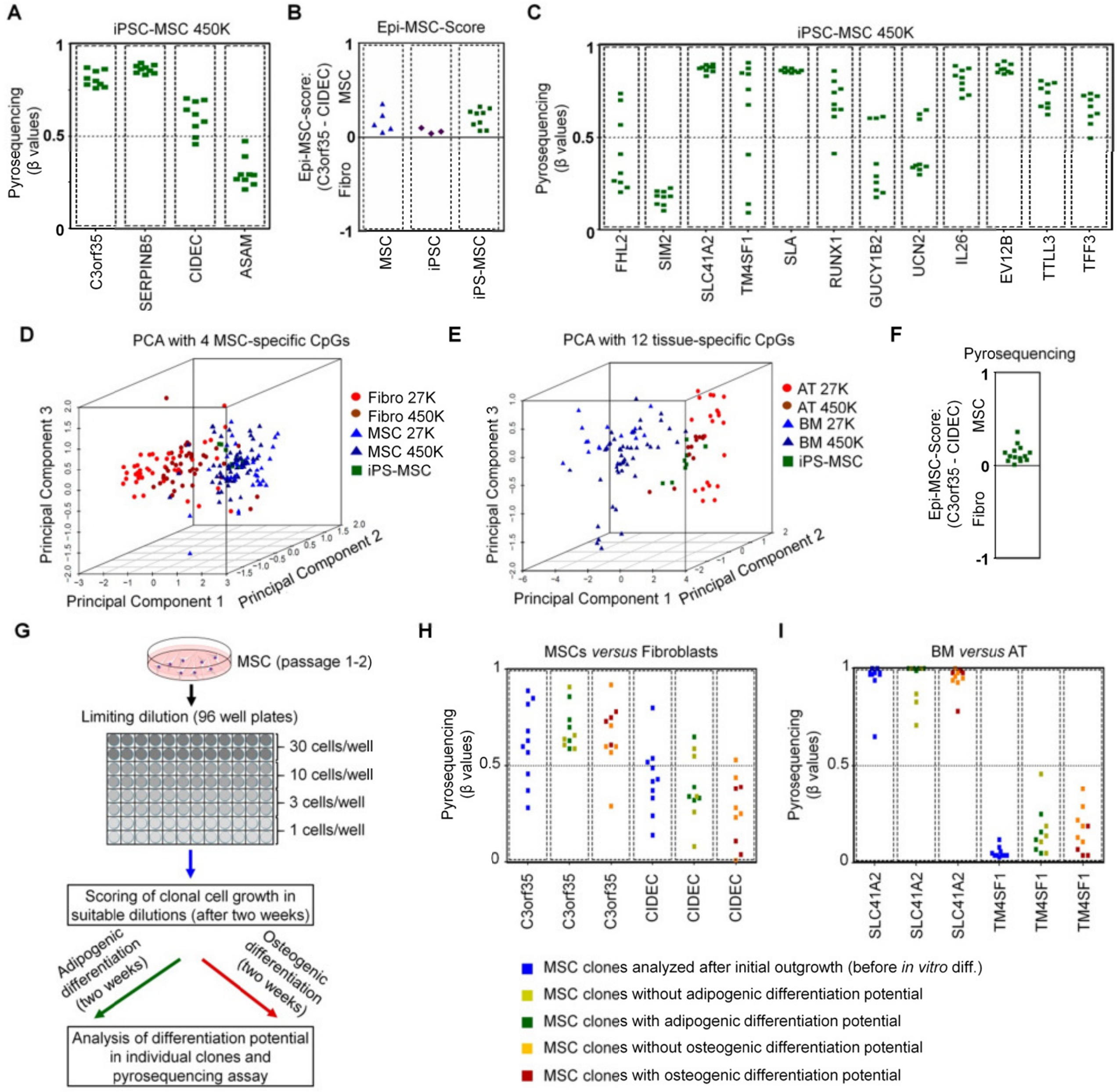

Figure S4. Classification of iPS-MSCs and of MSC clones, related to Figure 2, 3 and 4.

(A) DNAm levels of iPS-MSCs at four CpGs that can discern MSCs and fibroblasts. DNAm levels at the CpGs in *C3orf35*, *SERPINB5* and *ASAM* are overall similar to those in MSCs.

(B) The Epi-MSC-Score was used to classify parental MSCs that have been used for reprogramming, iPSCs, and iPS-MSCs. iPS-MSCs are rather classified as MSCs than as fibroblasts.

(C) DNAm levels of iPS-MSCs at 12 CpG-sites that are indicative for MSCs from bone marrow (BM) or adipose tissue (AT). This pattern did not clearly reflect bone marrow origin of iPS-MSCs.

(D) Principal component analysis (PCA) of the four MSC/fibroblast-associated CpGs (indicated in A) can clearly discern MSCs and fibroblasts. The results supported the notion that iPS-MSCs are related to MSCs.

(E) PCA of the 12 tissue associated CpGs (indicated in B) can clearly discern MSCs from bone marrow and adipose tissue. iPS-MSCs were not clearly associated to either BM- or AT-derived MSCs.

(F) Epi-MSC-Score analysis of iPS-MSCs by pyrosequencing demonstrated reproducible classification of independent clones as MSCs.

(G) Schematic representation of limiting dilutions of bone marrow MSCs and their *in vitro* differentiation. Dilutions were performed for three biological replica and only those dilutions were further considered that statistically correspond to single-cell derived colonies (Schellenberg et al., 2012). Each dilution was performed in triplicate to be (i) analyzed after two weeks without differentiation (blue), (ii) further differentiated for additional two weeks towards adipogenic lineage (green), and (iii) further differentiated towards osteogenic lineage (red). The cell clones (and differentiation potential) were scored by microscopy and absorbance measurement and DNA was then harvested for pyrosequencing analysis of the epigenetic scores.

(H) Pyrosequencing results of DNAm levels at the two CpGs of the Epi-MSC-Score.

(I) Pyrosequencing results of DNAm levels at the two CpGs of the Epi-Tissue-Score.

Table S1. DNAm profiles of the training dataset (27K BeadChip)

GEO-number	Cell-Type	Source	Location	Gender	Age	Passage	Serum	References
**GSE17448**	MSC	BM	IC	-	>40	P<5	FBS	(Bork et al., 2010)
**GSE17448**	MSC	BM	CF	-	>40	P<5	FBS	(Bork et al., 2010)
**GSE17448**	MSC	BM	CF	-	>40	P<5	FBS	(Bork et al., 2010)
**GSE17448**	MSC	BM	CF	-	>40	P<5	FBS	(Bork et al., 2010)
**GSE17448**	MSC	BM	CF	-	>40	P<5	FBS	(Bork et al., 2010)
**GSE17448**	MSC	BM	CF	-	>40	P>5	FBS	(Bork et al., 2010)
**GSE17448**	MSC	BM	IC	-	>40	P>5	FBS	(Bork et al., 2010)
**GSE17448**	MSC	BM	CF	-	>40	P>5	FBS	(Bork et al., 2010)
**GSE17448**	MSC	BM	CF	-	>40	P>5	FBS	(Bork et al., 2010)
**GSE17448**	MSC	BM	CF	-	>40	P>5	FBS	(Bork et al., 2010)
**GSE17448**	MSC	BM	IC	-	<40	P<5	FBS	(Bork et al., 2010)
**GSE17448**	MSC	BM	IC	-	<40	P<5	FBS	(Bork et al., 2010)
**GSE17448**	MSC	BM	IC	-	<40	P<5	FBS	(Bork et al., 2010)
**GSE17448**	MSC	BM	IC	-	<40	P>5	FBS	(Bork et al., 2010)
**GSE17448**	MSC	BM	IC	-	<40	P>5	FBS	(Bork et al., 2010)
**GSE17448**	MSC	BM	IC	-	<40	P>5	FBS	(Bork et al., 2010)
**GSE26519**	MSC	AT	-	F	<40	P<5	hPL	(Schellenberg et al., 2011)
**GSE26519**	MSC	AT	-	F	>40	P>5	hPL	(Schellenberg et al., 2011)
**GSE26519**	MSC	AT	-	M	>40	P>5	hPL	(Schellenberg et al., 2011)
**GSE26519**	MSC	AT	-	F	>40	P>5	hPL	(Schellenberg et al., 2011)
**GSE26519**	MSC	AT	-	F	<40	P>5	hPL	(Schellenberg et al., 2011)
**GSE26519**	MSC	AT	-	F	>40	P>5	hPL	(Schellenberg et al., 2011)
**GSE26519**	MSC	AT	-	F	>40	P>5	hPL	(Schellenberg et al., 2011)
**GSE26519**	MSC	AT	-	F	>40	P>5	hPL	(Schellenberg et al., 2011)
**GSE29661**	MSC	AT	Breast	F	>40	P<5	hPL	(Koch et al., 2012)
**GSE29661**	MSC	AT	Leg	F	>40	P<5	hPL	(Koch et al., 2012)
**GSE29661**	MSC	AT	Leg	F	>40	P>5	hPL	(Koch et al., 2012)
**GSE29661**	MSC	AT	Breast	F	>40	P>5	hPL	(Koch et al., 2012)
**GSE29873**	MSC	BM	-	-	-	-	-	(Ohm et al., 2010)
**GSE29873**	MSC	BM	-	-	-	-	-	(Ohm et al., 2010)
**GSE33896**	MSC	AT	-	-	>40	P>5	FBS	(Berdasco et al., 2012)
**GSE33896**	MSC	AT	-	-	>40	P>5	FBS	(Berdasco et al., 2012)
**GSE33896**	MSC	AT	-	-	>40	P>5	FBS	(Berdasco et al., 2012)
**GSE33896**	MSC	AT	-	-	>40	P>5	FBS	(Berdasco et al., 2012)
**GSE44222**	MSC	AT	-	-	-	-	-	(Sempere et al., 2014)
**GSE44222**	MSC	AT	-	-	-	-	-	(Sempere et al., 2014)
**GSE44222**	MSC	AT	-	-	-	-	-	(Sempere et al., 2014)
**GSE44222**	MSC	AT	-	-	-	-	-	(Sempere et al., 2014)
**GSE44222**	MSC	AT	-	-	-	-	-	(Sempere et al., 2014)
**GSE22595**	Fibroblast	Derm	Skin	F	>40	P<5	FBS	(Koch et al., 2011)
**GSE22595**	Fibroblast	Derm	Skin	F	>40	P<5	FBS	(Koch et al., 2011)
**GSE22595**	Fibroblast	Derm	Skin	F	>40	P<5	FBS	(Koch et al., 2011)
**GSE22595**	Fibroblast	Derm	Skin	F	>40	P<5	FBS	(Koch et al., 2011)
**GSE22595**	Fibroblast	Derm	Skin	F	>40	P<5	FBS	(Koch et al., 2011)
**GSE22595**	Fibroblast	Derm	Skin	F	>40	P<5	FBS	(Koch et al., 2011)
**GSE22595**	Fibroblast	Derm	Skin	F	<40	P<5	FBS	(Koch et al., 2011)
**GSE22595**	Fibroblast	Derm	Skin	F	<40	P<5	FBS	(Koch et al., 2011)
**GSE22595**	Fibroblast	Derm	Skin	F	<40	P<5	FBS	(Koch et al., 2011)
**GSE22595**	Fibroblast	Derm	Skin	F	<40	P<5	FBS	(Koch et al., 2011)
**GSE22595**	Fibroblast	Derm	Skin	F	<40	P<5	FBS	(Koch et al., 2011)
**GSE22595**	Fibroblast	Derm	Skin	F	<40	P<5	FBS	(Koch et al., 2011)
**GSE22595**	Fibroblast	Derm	Skin	F	<40	P<5	FBS	(Koch et al., 2011)
**GSE22595**	Fibroblast	Derm	Skin	F	<40	P<5	FBS	(Koch et al., 2011)
**GSE22595**	Fibroblast	Derm	Skin	F	<40	P>5	FBS	(Koch et al., 2011)
**GSE22874**	Fibroblast	Lung	-	F	>40	-	-	(Navab et al., 2011)

**GSE22874**	Fibroblast	Lung	-	M	>40	-	-	(Navab et al., 2011)
**GSE22874**	Fibroblast	Lung	-	M	>40	-	-	(Navab et al., 2011)
**GSE22874**	Fibroblast	Lung	-	F	>40	-	-	(Navab et al., 2011)
**GSE22874**	Fibroblast	Lung	-	M	>40	-	-	(Navab et al., 2011)
**GSE24676**	Fibroblast	Lung	-	M	-	-	FBS	(Nishino et al., 2011)
**GSE29661**	Fibroblast	Derm	Skin	F	>40	P<5	FBS	(Koch et al., 2012)
**GSE29661**	Fibroblast	Derm	Skin	F	>40	P>5	FBS	(Koch et al., 2012)
**GSE29661**	Fibroblast	Derm	Skin	F	>40	P<5	FBS	(Koch et al., 2012)
**GSE29661**	Fibroblast	Derm	Skin	F	>40	P<5	FBS	(Koch et al., 2012)
**GSE29661**	Fibroblast	Derm	Skin	F	>40	P<5	FBS	(Koch et al., 2012)
**GSE29661**	Fibroblast	Derm	Skin	F	>40	P>5	FBS	(Koch et al., 2012)
**GSE29661**	Fibroblast	Derm	Skin	F	>40	P>5	FBS	(Koch et al., 2012)
**GSE29661**	Fibroblast	Derm	Skin	F	>40	P>5	FBS	(Koch et al., 2012)
**GSE29873**	Fibroblast	Lung	-	-	-	-	-	(Ohm et al., 2010)
**GSE30640**	Fibroblast	Lung	-	-	-	P>5	-	-
**GSE30640**	Fibroblast	Lung	-	-	-	P>5	-	-
**GSE30640**	Fibroblast	Lung	-	-	-	P>5	-	-
**GSE30640**	Fibroblast	Lung	-	-	-	P>5	-	-
**GSE30640**	Fibroblast	Lung	-	-	-	P>5	-	-
**GSE42043**	Fibroblast	Skin	-	M	-	P<5	-	(Huang et al., 2014)
**GSE42043**	Fibroblast	Skin	-	M	-	P<5	-	(Huang et al., 2014)
**GSE42043**	Fibroblast	Lung	-	F	-	P>5	-	(Huang et al., 2014)
**GSE42043**	Fibroblast	Lung	-	F	-	P>5	-	(Huang et al., 2014)
**GSE42043**	Fibroblast	-	-	F	-	P<5	-	(Huang et al., 2014)
**GSE42043**	Fibroblast	Skin	-	M	-	P<5	-	(Huang et al., 2014)
**GSE42043**	Fibroblast	-	-	M	-	P<5	-	(Huang et al., 2014)
**GSE42043**	Fibroblast	Skin	-	M	-	P<5	-	(Huang et al., 2014)
**GSE49053**	Fibroblast	Derm	Skin	-	-	-	FBS	(Koyanagi-Aoi et al., 2013)

F = female; M = male; P = passage; FBS = fetal bovine serum and hPL = human platelet lysate; IC = iliac crest; CF = caput femoris.

Table S2. DNAm profiles of the validation dataset (450K BeadChip)

GEO-number	Cell-Type	Source	Location	Gender	Age	Passage	Serum	References
**GSE55571**	MSC	BM	-	-	-	-	FBS	(Miyata et al., 2015)
**GSE34688**	MSC	BM	-	-	-	P<5	hPL	(Shao et al., 2013)
**GSE34688**	MSC	BM	-	-	-	P<5	hPL	(Shao et al., 2013)
**GSE34688**	MSC	BM	-	-	-	P<5	hPL	(Shao et al., 2013)
**GSE34688**	MSC	BM	-	-	-	P<5	hPL	(Shao et al., 2013)
**GSE34688**	MSC	BM	-	-	-	P<5	hPL	(Shao et al., 2013)
**GSE37067**	MSC	BM	-	-	-	P<5	hPL	(Koch et al., 2013)
**GSE37067**	MSC	BM	-	-	-	P<5	hPL	(Koch et al., 2013)
**GSE37067**	MSC	BM	-	-	-	P<5	hPL	(Koch et al., 2013)
**GSE37067**	MSC	BM	-	-	-	P<5	hPL	(Koch et al., 2013)
**GSE37067**	MSC	BM	-	-	-	P<5	hPL	(Koch et al., 2013)
**GSE37067**	MSC	BM	-	-	-	P>5	hPL	(Koch et al., 2013)
**GSE37067**	MSC	BM	-	-	-	P>5	hPL	(Koch et al., 2013)
**GSE37067**	MSC	BM	-	-	-	P>5	hPL	(Koch et al., 2013)
**GSE37067**	MSC	BM	-	-	-	P>5	hPL	(Koch et al., 2013)
**GSE52114**	MSC	BM	-	-	<40	P>5	FBS	(Fernandez et al., 2015)
**GSE52114**	MSC	BM	-	-	<40	P>5	FBS	(Fernandez et al., 2015)
**GSE52114**	MSC	BM	-	-	<40	P>5	FBS	(Fernandez et al., 2015)
**GSE52114**	MSC	BM	-	-	<40	P>5	FBS	(Fernandez et al., 2015)
**GSE52114**	MSC	BM	-	-	<40	P>5	FBS	(Fernandez et al., 2015)
**GSE52114**	MSC	BM	-	-	<40	P>5	FBS	(Fernandez et al., 2015)
**GSE52114**	MSC	BM	-	-	<40	P>5	FBS	(Fernandez et al., 2015)
**GSE52114**	MSC	BM	-	-	<40	P>5	FBS	(Fernandez et al., 2015)
**GSE52114**	MSC	BM	-	-	<40	P>5	FBS	(Fernandez et al., 2015)
**GSE52114**	MSC	BM	-	-	<40	P>5	FBS	(Fernandez et al., 2015)
**GSE52114**	MSC	BM	-	-	<40	P>5	FBS	(Fernandez et al., 2015)
**GSE52114**	MSC	BM	-	-	<40	P>5	FBS	(Fernandez et al., 2015)
**GSE52114**	MSC	BM	-	-	<40	P>5	FBS	(Fernandez et al., 2015)
**GSE52114**	MSC	BM	-	-	<40	P>5	FBS	(Fernandez et al., 2015)
**GSE52114**	MSC	BM	-	-	<40	P>5	FBS	(Fernandez et al., 2015)
**GSE52114**	MSC	BM	-	-	<40	P>5	FBS	(Fernandez et al., 2015)
**GSE52114**	MSC	BM	-	-	<40	P>5	FBS	(Fernandez et al., 2015)
**GSE52114**	MSC	BM	-	-	<40	P>5	FBS	(Fernandez et al., 2015)
**GSE52114**	MSC	BM	-	-	<40	P>5	FBS	(Fernandez et al., 2015)
**GSE52114**	MSC	BM	-	-	<40	P>5	FBS	(Fernandez et al., 2015)
**GSE52114**	MSC	BM	-	-	<40	P>5	FBS	(Fernandez et al., 2015)
**GSE52114**	MSC	BM	-	-	<40	P>5	FBS	(Fernandez et al., 2015)
**GSE52114**	MSC	BM	-	-	>40	P>5	FBS	(Fernandez et al., 2015)
**GSE52114**	MSC	BM	-	-	>40	P>5	FBS	(Fernandez et al., 2015)
**GSE52114**	MSC	BM	-	-	>40	P>5	FBS	(Fernandez et al., 2015)
**GSE52114**	MSC	BM	-	-	>40	P>5	FBS	(Fernandez et al., 2015)
**GSE52114**	MSC	BM	-	-	>40	P>5	FBS	(Fernandez et al., 2015)
**GSE52114**	MSC	BM	-	-	>40	P>5	FBS	(Fernandez et al., 2015)
**GSE52114**	MSC	BM	-	-	>40	P>5	FBS	(Fernandez et al., 2015)
**GSE52114**	MSC	BM	-	-	>40	P>5	FBS	(Fernandez et al., 2015)
**GSE52114**	MSC	BM	-	-	>40	P>5	FBS	(Fernandez et al., 2015)
**GSE52114**	MSC	BM	-	-	>40	P>5	FBS	(Fernandez et al., 2015)
**GSE52114**	MSC	BM	-	-	>40	P>5	FBS	(Fernandez et al., 2015)
**GSE52114**	MSC	BM	-	-	>40	P>5	FBS	(Fernandez et al., 2015)
**GSE55889**	MSC	AT	-	-	-	P<5	hPL	(Schellenberg et al., 2014)
**GSE55889**	MSC	AT	-	-	-	P<5	hPL	(Schellenberg et al., 2014)
**GSE55889**	MSC	AT	-	-	-	P<5	hPL	(Schellenberg et al., 2014)
**GSE55889**	MSC	AT	-	-	-	P<5	hPL	(Schellenberg et al., 2014)
**GSE55889**	MSC	AT	-	-	-	P<5	hPL	(Schellenberg et al., 2014)
**GSE55889**	MSC	AT	-	-	-	P<5	hPL	(Schellenberg et al., 2014)

**GSE55889**	MSC	AT	-	-	-	P<5	hPL	(Schellenberg et al., 2014)
**GSE55889**	MSC	AT	-	-	-	P<5	hPL	(Schellenberg et al., 2014)
**GSE57151**	MSC	AT	-	-	-	-	hPL	(Reinisch et al., 2015)
**GSE57151**	MSC	AT	-	-	-	-	hPL	(Reinisch et al., 2015)
**GSE57151**	MSC	AT	-	-	-	-	hPL	(Reinisch et al., 2015)
**GSE57151**	MSC	UC	-	-	-	-	hPL	(Reinisch et al., 2015)
**GSE57151**	MSC	UC	-	-	-	-	hPL	(Reinisch et al., 2015)
**GSE57151**	MSC	UC	-	-	-	-	hPL	(Reinisch et al., 2015)
**GSE57151**	MSC	BM	-	-	-	-	hPL	(Reinisch et al., 2015)
**GSE57151**	MSC	BM	-	-	-	-	hPL	(Reinisch et al., 2015)
**GSE57151**	MSC	BM	-	-	-	-	hPL	(Reinisch et al., 2015)
**GSE30654**	Fibroblast	Skin	-	-	-	-	FBS	(Nazor et al., 2012)
**GSE30654**	Fibroblast	Lung	-	-	-	-	FBS	(Nazor et al., 2012)
**GSE30654**	Fibroblast	Derm	-	-	-	-	FBS	(Nazor et al., 2012)
**GSE30654**	Fibroblast	Derm	-	-	-	-	FBS	(Nazor et al., 2012)
**GSE30654**	Fibroblast	Derm	-	-	-	-	FBS	(Nazor et al., 2012)
**GSE30654**	Fibroblast	Derm	-	-	-	-	FBS	(Nazor et al., 2012)
**GSE30654**	Fibroblast	Heart	-	-	-	-	FBS	(Nazor et al., 2012)
**GSE30654**	Fibroblast	Heart	-	-	-	-	FBS	(Nazor et al., 2012)
**GSE30654**	Fibroblast	Lung	-	-	-	-	FBS	(Nazor et al., 2012)
**GSE40790**	Fibroblast	Lung	-	-	-	-	FBS	(Merling et al., 2013)
**GSE40927**	Fibroblast	Skin	-	-	-	P<5	FBS	(Kurian et al., 2013)
**GSE40927**	Fibroblast	Skin	-	-	-	P<5	FBS	(Kurian et al., 2013)
**GSE40927**	Fibroblast	Skin	-	-	-	P>5	FBS	(Kurian et al., 2013)
**GSE46650**	Fibroblast	Synovial	-	-	-	-	FBS	(de la Rica et al., 2013)
**GSE46650**	Fibroblast	Synovial	-	-	-	-	FBS	(de la Rica et al., 2013)
**GSE46650**	Fibroblast	Synovial	-	-	-	-	FBS	(de la Rica et al., 2013)
**GSE46650**	Fibroblast	Synovial	-	-	-	-	FBS	(de la Rica et al., 2013)
**GSE46650**	Fibroblast	Synovial	-	-	-	-	FBS	(de la Rica et al., 2013)
**GSE46650**	Fibroblast	Synovial	-	-	-	-	FBS	(de la Rica et al., 2013)
**GSE46650**	Fibroblast	Synovial	-	-	-	-	FBS	(de la Rica et al., 2013)
**GSE46650**	Fibroblast	Synovial	-	-	-	-	FBS	(de la Rica et al., 2013)
**GSE46650**	Fibroblast	Synovial	-	-	-	-	FBS	(de la Rica et al., 2013)
**GSE46650**	Fibroblast	Synovial	-	-	-	-	FBS	(de la Rica et al., 2013)
**GSE46650**	Fibroblast	Synovial	-	-	-	-	FBS	(de la Rica et al., 2013)
**GSE46650**	Fibroblast	Synovial	-	-	-	-	FBS	(de la Rica et al., 2013)
**GSE53096**	Fibroblast	Derm	-	-	-	-	FBS	(Ma et al., 2014)
**GSE53918**	Fibroblast	Cornea	-	-	-	-	FBS	(Sareen et al., 2014)
**GSE53918**	Fibroblast	Cornea	-	-	-	-	FBS	(Sareen et al., 2014)
**GSE53918**	Fibroblast	Cornea	-	-	-	-	FBS	(Sareen et al., 2014)
**GSE54115**	Fibroblast	Skin	-	-	-	P>5	FBS	-
**GSE54115**	Fibroblast	Skin	-	-	-	P>5	FBS	-
**GSE54848**	Fibroblast	Derm	-	-	-	-	FBS	(Ohnuki et al., 2014)
**GSE54848**	Fibroblast	Derm	-	-	-	-	FBS	(Ohnuki et al., 2014)
**GSE54848**	Fibroblast	Derm	-	-	-	-	FBS	(Ohnuki et al., 2014)
**GSE57151**	Fibroblast	-	-	-	-	-	FBS	(Reinisch et al., 2015)
**GSE57151**	Fibroblast	-	-	-	-	-	FBS	(Reinisch et al., 2015)
**GSE57151**	Fibroblast	-	-	-	-	-	FBS	(Reinisch et al., 2015)
**GSE57992**	Fibroblast	Aminiotic	-	-	-	-	FBS	(He et al., 2014)
**GSE57992**	Fibroblast	-	-	-	-	-	FBS	(He et al., 2014)
**GSE61461**	Fibroblast	Skin	-	-	-	-	FBS	(Johannesson et al., 2014)
**GSE61461**	Fibroblast	Skin	-	-	-	-	FBS	(Johannesson et al., 2014)

P = passage; FBS = fetal bovine serum; hPL = human platelet lysate; IC = iliac crest; CF = caput femoris; UC = umbilical cord.

Table S3. Primary cell preparations used for pyrosequencing analysis

Sample ID	Tissue Source	Age	Gender	Passage
MSC 1	Bone Marrow	69	Female	3
MSC 2	Bone Marrow	84	Male	2
MSC 3	Bone Marrow	50	Female	1
MSC 4	Bone Marrow	33	Female	2
MSC 5	Bone Marrow	59	Male	1
MSC 6	Bone Marrow	50	Male	2
MSC 7	Bone Marrow	30	Male	2
MSC 8	Bone Marrow	70	Female	2
MSC 9	Bone Marrow	87	Male	1
MSC 10	Bone Marrow	73	Male	1
MSC 11	Bone Marrow	66	Male	1
MSC 12	Bone Marrow	67	Male	2
MSC 13	Adipose Tissue	46	Male	4
MSC 14	Adipose Tissue	43	Female	2
MSC 15	Adipose Tissue	48	Female	2
MSC 16	Adipose Tissue	50	Female	3
MSC 17	Adipose Tissue	43	Female	1
MSC 18	Adipose Tissue	19	Female	3
MSC 19	Adipose Tissue	24	Male	1
MSC 20	Adipose Tissue	23	Male	1
MSC 21	Adipose Tissue	24	Female	1
MSC 22	Adipose Tissue	29	Female	2
Fibroblast 1	Dermis (Breast)	42	Female	2
Fibroblast 2	Dermis (Abdomen)	62	Female	2
Fibroblast 3	Dermis (Breast)	43	Female	2
Fibroblast 4	Dermis (Breast)	18	Female	2
Fibroblast 5	Dermis (Arm)	63	Female	2
Fibroblast 6	Dermis (Abdomen)	73	Female	2
Fibroblast 7	Dermis (Ear)	64	Female	2
Fibroblast 8	Dermis (Breast)	60	Female	3
Fibroblast 9	Dermis (Abdomen)	23	Female	11
Fibroblast 10	Dermis	60	Female	10
Fibroblast 11	Dermis	60	Female	5
Fibroblast 12	Dermis (Breast)	60	Female	16

Table S4. Primers for pyrosequencing assays

Primer	CpG ID	Sequence
C3orf35_F Biotin	cg22286764	5`-TGTGTGTATTTTGTTGTTTATTTTTTGGGTTTAGGAGAA-3`
C3orf35_R		5`-CCTCCCTTAAAATCAATCTCCAATCATTTAATTAACTT-3`
C3orf35_seq		5`-AACTTAACTACAATCATTCACA-3`
CIDEC_F	cg05684195	5`-TGAGTAGATAATTTAATTTAGGGTTGAAGAAGTTTTGT-3`
CIDEC _R Biotin		5`-CATCCCCAAAAATATAAAATAATATAACTCACCTCC-3`
CIDEC_seq		5`-AGATTTGTTTTTGTTTATGG-3`
SLC41A2_F	cg27149093	5`-TGTGTTTGTTTTTTTGGTTTTTTTGGTAAATTA-3`
SLC41A2_R Biotin		5`-CCAAATCCAATCCTTCTATAAAACTTCTAAACATCTT-3`
SLC41A2_seq		5`-GGGAATTATAGTAGATGAATTAG-3`
TM4SF1_F Biotin	cg08124030	5`-ATAAAGAGAAGGGGGAGAAAATTTAGTAGATTATTATGTG-3`
TM4SF1_R		5`-TTTCCCCATTAAAAAAATAAAACAAAATATTAACC-3`
TM4SF1_seq		5`-AAAAATAAAACAAAATATTAACC-3`

_F = forward primer; _R = reverse primer.

Supplemental Experimental Procedures

Bioinformatic procedures

The DNAm datasets (Supplemental Tables S1 and S2) were carefully evaluated with regard to the corresponding publications. CpGs linked to X and Y chromosomes were excluded and we focused on 25,014 CpGs represented by 27K and 450K BeadChips. DNAm levels (β-values) were quantile normalized using the R package *lumi*. Principal component analysis (PCA) was performed with the R package *stats* using singular value decomposition of the data matrix. Significant differentially methylated CpG-sites were identified by Limma adjusted t-test (R package *limma*; $p < 0.05$).

Permutation assays

Bootstrapping was performed on the 27k BeadChip training set to estimate likelihood that the CpGs of the Epi-MSC-Score provide reproducible results according to (i) difference in mean DNAm in MSCs *versus* fibroblasts, and (ii) variation in DNAm levels within each of the two cell types. This method was performed 1000 times. The results revealed that the CpG site in *CIDEC* (cg05684195) was on rank four (88% of replicates) and the CpG site in *C3orf35* (cg22286764) was on rank eight (73% of replicates) of all 25,014 CpGs. In analogy, we performed the same experiment for the DNAm changes between MSCs from BM *versus* AT: *TM4SF1* (cg08124030) was on rank one (100% of the replicates) and *SLC41A2* (cg27149093) was on rank nine (93% of the replicates). This reanalysis supported the notion that the CpGs of the Epi-MSC-Score and of the Epi-Tissue-Score are within the most stable CpGs sites for these comparisons.

Analysis of Epi-MSC-Score in other cell types

DNAm profiles (450k) of MSCs that were subsequently used for reprogramming into iPSCs (GSE34688), of iPSCs (GSE34688), and of iPS-MSCs (GSE54767) were retrieved from GEO. Furthermore, we applied the Epi-MSC-Score to DNAm profiles of blood (GSE39981), monocytes (GSE59339), and macrophages (GSE31680). Overall, Epi-MSC-Score of these hematopoietic cells was close to zero, indicating that potentially contaminating macrophages would hardly impact on the Epi-MSC-Score – in this regard the Epi-MSC-Score is no substitute for immunophenotypic analysis.

Analysis of differential gene expression in genes of Epi-MSC-Score and Epi-Tissue-Score

To estimate if DNAm changes might be reflected in differential gene expression we downloaded microarray data from GEO (all Illumina HumanHT-12 v4 platform; GPL10558). For analysis of gene expression of *C3orf35* and *CIDEC* (associated with Epi-MSC-Score) in MSCs *versus* fibroblasts we used the following profiles: for MSCs: GSM1050328, GSM1050329, GSM1050330, GSM1050331, GSM1050332, GSM1050333, GSM1128574, GSM1128575, GSM1276944, GSM1276947, GSM1276948, GSM1276949, GSM1350082, GSM1350083, GSM1350088, GSM1350089, GSM1350090, GSM1515746, GSM1515747, GSM1515748, GSM1515749, GSM1515750, GSM1515751, GSM1515752; and for fibroblasts: GSM786856, GSM786857, GSM1348171, GSM1348172, GSM1348173, GSM860982, GSM860983, GSM860984, GSM1359297, GSM1359298, GSM1359309, GSM1359310, GSM1381443, GSM1586080, GSM1586082, GSM1586085, GSM1586089, GSM1329667, GSM1329668, GSM1664886, GSM1664890, GSM1664894. To estimate gene expression levels of *SLC41A2* and *TM4SF1* (associated with CpGs of the Epi-Tissue-Score) we utilized the following profiles for MSCs from bone marrow: GSM1050328, GSM1050329, GSM1050330, GSM1050331, GSM1050332, GSM1050333, GSM1128574, GSM1128575, GSM1276944, GSM1276947, GSM1276948, GSM1276949, GSM1350082, GSM1350083, GSM1350088, GSM1350089, GSM1350090; and for MSCs from adipose tissue: GSM1515746, GSM1515747, GSM1515748, GSM1515749, GSM1515750, GSM1515751, GSM1515752. Data were quantile normalized for comparison of relative gene expression levels.

Additional information on clonal analysis of MSCs

After two weeks, individual clones with confluence of 50% or more were counted to estimate the *CFU-Fs* (colony-forming unit fibroblast-like) frequency with the *L-Calc Software* (Stem Cell Technologies, Canada) (Schellenberg et al., 2012) and then harvested. In addition, we used independent 96-well plates, that were either differentiated towards osteogenic or adipogenic lineages for two additional weeks and stained with Alizarin Red or BODIPY, respectively (Schellenberg et al., 2012). After absorbance measurement (Tecan PRO, Switzerland) and fluorescence analysis (EVOS, Life Technologies, USA) the DNA was harvested for pyrosequencing.

Supplemental References:

Berdasco, M., Melguizo, C., Prados, J., Gomez, A., Alaminos, M., Pujana, M.A., Lopez, M., Setien, F., Ortiz, R., Zafra, I., *et al.* (2012). DNA methylation plasticity of human adipose-derived stem cells in lineage commitment. Am J Pathol *181*, 2079-2093.

Bork, S., Pfister, S., Witt, H., Horn, P., Korn, B., Ho, A.D., and Wagner, W. (2010). DNA methylation pattern changes upon long-term culture and aging of human mesenchymal stromal cells. Aging cell *9*, 54-63.

de la Rica, L., Urquiza, J.M., Gomez-Cabrero, D., Islam, A.B., Lopez-Bigas, N., Tegner, J., Toes, R.E., and Ballestar, E. (2013). Identification of novel markers in rheumatoid arthritis through integrated analysis of DNA methylation and microRNA expression. J Autoimmun *41*, 6-16.

Fernandez, A.F., Bayon, G.F., Urdinguio, R.G., Torano, E.G., Garcia, M.G., Carella, A., Petrus-Reurer, S., Ferrero, C., Martinez-Camblor, P., Cubillo, I., *et al.* (2015). H3K4me1 marks DNA regions hypomethylated during aging in human stem and differentiated cells. Genome Res *25*, 27-40.

He, W., Kang, X., Du, H., Song, B., Lu, Z., Huang, Y., Wang, D., Sun, X., Yu, Y., and Fan, Y. (2014). Defining differentially methylated regions specific for the acquisition of pluripotency and maintenance in human pluripotent stem cells via microarray. PloS one *9*, e108350.

Huang, K., Shen, Y., Xue, Z., Bibikova, M., April, C., Liu, Z., Cheng, L., Nagy, A., Pellegrini, M., Fan, J.B., *et al.* (2014). A panel of CpG methylation sites distinguishes human embryonic stem cells and induced pluripotent stem cells. Stem Cell Reports *2*, 36-43.

Johannesson, B., Sagi, I., Gore, A., Paull, D., Yamada, M., Golan-Lev, T., Li, Z., LeDuc, C., Shen, Y., Stern, S., *et al.* (2014). Comparable frequencies of coding mutations and loss of imprinting in human pluripotent cells derived by nuclear transfer and defined factors. Cell Stem Cell *15*, 634-642.

Koch, C.M., Joussen, S., Schellenberg, A., Lin, Q., Zenke, M., and Wagner, W. (2012). Monitoring of cellular senescence by DNA-methylation at specific CpG-sites. Aging cell *11*, 366-369.

Koch, C.M., Reck, K., Shao, K., Lin, Q., Joussen, S., Ziegler, P., Walenda, G., Drescher, W., Opalka, B., May, T., *et al.* (2013). Pluripotent stem cells escape from senescence-associated DNA methylation changes. Genome Res *23*, 248-259.

Koch, C.M., Suschek, C.V., Lin, Q., Bork, S., Goergens, M., Joussen, S., Pallua, N., Ho, A.D., Zenke, M., and Wagner, W. (2011). Specific age-associated DNA methylation changes in human dermal fibroblasts. PloS one *6*, e16679.

Koyanagi-Aoi, M., Ohnuki, M., Takahashi, K., Okita, K., Noma, H., Sawamura, Y., Teramoto, I., Narita, M., Sato, Y., Ichisaka, T., *et al.* (2013). Differentiation-defective phenotypes revealed by large-scale analyses of human pluripotent stem cells. Proc Natl Acad Sci U S A *110*, 20569-20574.

Kurian, L., Sancho-Martinez, I., Nivet, E., Aguirre, A., Moon, K., Pendaries, C., Volle-Challier, C., Bono, F., Herbert, J.M., Pulecio, J., *et al.* (2013). Conversion of human fibroblasts to angioblast-like progenitor cells. Nat Methods *10*, 77-83.

Ma, H., Morey, R., O'Neil, R.C., He, Y., Daughtry, B., Schultz, M.D., Hariharan, M., Nery, J.R., Castanon, R., Sabatini, K., *et al.* (2014). Abnormalities in human pluripotent cells due to reprogramming mechanisms. Nature *511*, 177-183.

Merling, R.K., Sweeney, C.L., Choi, U., De Ravin, S.S., Myers, T.G., Otaizo-Carrasquero, F., Pan, J., Linton, G., Chen, L., Koontz, S., *et al.* (2013). Transgene-free iPSCs generated from small volume peripheral blood nonmobilized CD34+ cells. Blood *121*, e98-107.

Miyata, K., Miyata, T., Nakabayashi, K., Okamura, K., Naito, M., Kawai, T., Takada, S., Kato, K., Miyamoto, S., Hata, K., *et al.* (2015). DNA methylation analysis of human myoblasts during in vitro myogenic differentiation: de novo methylation of promoters of muscle-related genes and its involvement in transcriptional down-regulation. Hum Mol Genet *24*, 410-423.

Navab, R., Strumpf, D., Bandarchi, B., Zhu, C.Q., Pintilie, M., Ramnarine, V.R., Ibrahimov, E., Radulovich, N., Leung, L., Barczyk, M., *et al.* (2011). Prognostic gene-expression signature of carcinoma-associated fibroblasts in non-small cell lung cancer. Proc Natl Acad Sci U S A *108*, 7160-7165.

Nazor, K.L., Altun, G., Lynch, C., Tran, H., Harness, J.V., Slavin, I., Garitaonandia, I., Muller, F.J., Wang, Y.C., Boscolo, F.S., *et al.* (2012). Recurrent variations in DNA methylation in human pluripotent stem cells and their differentiated derivatives. Cell Stem Cell *10*, 620-634.

Nishino, K., Toyoda, M., Yamazaki-Inoue, M., Fukawatase, Y., Chikazawa, E., Sakaguchi, H., Akutsu, H., and Umezawa, A. (2011). DNA methylation dynamics in human induced pluripotent stem cells over time. PLoS Genet *7*, e1002085.

Ohm, J.E., Mali, P., Van Neste, L., Berman, D.M., Liang, L., Pandiyan, K., Briggs, K.J., Zhang, W., Argani, P., Simons, B., *et al.* (2010). Cancer-related epigenome changes associated with reprogramming to induced pluripotent stem cells. Cancer Res *70*, 7662-7673.

Ohnuki, M., Tanabe, K., Sutou, K., Teramoto, I., Sawamura, Y., Narita, M., Nakamura, M., Tokunaga, Y., Nakamura, M., Watanabe, A., *et al.* (2014). Dynamic regulation of human endogenous retroviruses mediates factor-induced reprogramming and differentiation potential. Proc Natl Acad Sci U S A *111*, 12426-12431.

Reinisch, A., Etchart, N., Thomas, D., Hofmann, N.A., Fruehwirth, M., Sinha, S., Chan, C.K., Senarath-Yapa, K., Seo, E.Y., Wearda, T., *et al.* (2015). Epigenetic and in vivo comparison of diverse MSC sources reveals an endochondral signature for human hematopoietic niche formation. Blood *125*, 249-260.

Sareen, D., Saghizadeh, M., Ornelas, L., Winkler, M.A., Narwani, K., Sahabian, A., Funari, V.A., Tang, J., Spurka, L., Punj, V., *et al.* (2014). Differentiation of human limbal-derived induced pluripotent stem cells into limbal-like epithelium. Stem Cells Transl Med *3*, 1002-1012.

Schellenberg, A., Joussen, S., Moser, K., Hampe, N., Hersch, N., Hemeda, H., Schnitker, J., Denecke, B., Lin, Q., Pallua, N., *et al.* (2014). Matrix elasticity, replicative senescence and DNA methylation patterns of mesenchymal stem cells. Biomaterials *35*, 6351-6358.

Schellenberg, A., Lin, Q., Schuler, H., Koch, C.M., Joussen, S., Denecke, B., Walenda, G., Pallua, N., Suschek, C.V., Zenke, M., *et al.* (2011). Replicative senescence of mesenchymal stem cells causes DNA-methylation changes which correlate with repressive histone marks. Aging (Albany NY) *3*, 873-888.

Schellenberg, A., Stiehl, T., Horn, P., Joussen, S., Pallua, N., Ho, A.D., and Wagner, W. (2012). Population dynamics of mesenchymal stromal cells during culture expansion. Cytotherapy *14*, 401-411.

Sempere, J.M., Martinez-Peinado, P., Arribas, M.I., Reig, J.A., De La Sen, M.L., Zubcoff, J.J., Fraga, M.F., Fernandez, A.F., Santana, A., and Roche, E. (2014). Single cell-derived clones from human adipose stem cells present different immunomodulatory properties. Clin Exp Immunol *176*, 255-265.

Shao, K., Koch, C., Gupta, M.K., Lin, Q., Lenz, M., Laufs, S., Denecke, B., Schmidt, M., Linke, M., Hennies, H.C., *et al.* (2013). Induced pluripotent mesenchymal stromal cell clones retain donor-derived differences in DNA methylation profiles. Mol Ther *21*, 240-250.
