# Supplementary material for: Epigenetic Classification of Human Mesenchymal Stromal Cells
Source: Stem Cell Reports. 2016 Feb 9;6(2):168–75. doi: 10.1016/j.stemcr.2016.01.003 (PMC4750140; doi:10.1016/j.stemcr.2016.01.003)
Supplement: Document S2. Article plus Supplemental Information [file mmc2.pdf]

Stem Cell Reports
Report
OPEN ACCESS

Epigenetic Classification of Human Mesenchymal Stromal Cells

Danilo Candido de Almeida,[1,2,3] Marcelo R.P. Ferreira,[4,5] Julia Franzen,[1,2] Carola I. Weidner,[1,2] Joana Frobel,[1,2] Martin Zenke,[2,6] Ivan G. Costa,[4] and Wolfgang Wagner[1,2,*]
[1]Division of Stem Cell Biology and Cellular Engineering, Helmholtz-Institute for Biomedical Engineering, RWTH Aachen University Medical School, Pauwelsstraße 20, 52074 Aachen, Germany
[2]Department of Cell Biology, Institute for Biomedical Engineering, RWTH Aachen University Medical School, 52074 Aachen, Germany
[3]Department of Immunology, Institute of Biomedical Sciences, University of São Paulo, São Paulo 05508-000, Brazil
[4]Department of Cell Biology, IZKF Research Group Bioinformatics, Institute for Biomedical Engineering, RWTH Aachen University Medical School, 52074 Aachen, Germany
[5]Department of Statistics, Centre for Natural and Exact Sciences, Federal University of Paraiba, João Pessoa 58051-900, Brazil
[6]Helmholtz Institute for Biomedical Engineering, RWTH Aachen University, 52074 Aachen, Germany
*Correspondence: wwagner@ukaachen.de
http://dx.doi.org/10.1016/j.stemcr.2016.01.003
This is an open access article under the CC BY license (http://creativecommons.org/licenses/by/4.0/).

SUMMARY

Standardization of mesenchymal stromal cells (MSCs) is hampered by the lack of a precise definition for these cell preparations; for example, there are no molecular markers to discern MSCs and fibroblasts. In this study, we followed the hypothesis that specific DNA methylation (DNAm) patterns can assist classification of MSCs. We utilized 190 DNAm profiles to address the impact of tissue of origin, donor age, replicative senescence, and serum supplements on the epigenetic makeup. Based on this, we elaborated a simple epigenetic signature based on two CpG sites to classify MSCs and fibroblasts, referred to as the Epi-MSC-Score. Another two-CpG signature can distinguish between MSCs from bone marrow and adipose tissue, referred to as the Epi-Tissue-Score. These assays were validated by site-specific pyrosequencing analysis in 34 primary cell preparations. Furthermore, even individual subclones of MSCs were correctly classified by our epigenetic signatures. In summary, we propose an alternative concept to use DNAm patterns for molecular definition of cell preparations, and our epigenetic scores facilitate robust and cost-effective quality control of MSC cultures.

INTRODUCTION

Mesenchymal stromal cells (MSCs) are currently tested for a wide range of clinical applications (Squillaro et al., 2015), but there are no precise measures for their quality control. Molecular markers to clearly discern MSCs and fibroblasts remain elusive. The major difference between these two cell types is that particularly MSCs comprise a multipotent subset often referred to as "mesenchymal stem cells" (Dominici et al., 2006). Several surface markers have been suggested for enrichment of MSCs, such as CD106, CD146, and CD271 (Buhring et al., 2007; Halfon et al., 2011; Sorrentino et al., 2008), but none of them seems to be exclusively expressed on MSCs. Proteomics and gene-expression profiles can discern cells that have been obtained from different tissues or under different culture conditions (Holley et al., 2015; Ishii et al., 2005), and high-content screening assays based on microRNA or RNAi can elucidate cell type-specific responses (Bae et al., 2009; Erdmann et al., 2015). However, all these profiling and high-throughput techniques are relatively time and labor consuming, require complex computational analysis, and can hardly be standardized for quality control of MSC preparations.

Cellular differentiation is reflected by specific epigenetic patterns. DNA methylation (DNAm) is the best characterized epigenetic modification, where cytosine guanine dinucleotides (CpGs) are covalently methylated at the cytosine residue (Jaenisch and Bird, 2003). DNAm has several advantages as a biomarker for classification of cell preparations: (1) it is rather stable; (2) it facilitates quantitative analysis at single-nucleotide resolution, and (3) it is directly coupled to cellular differentiation (Karnik and Meissner, 2013). We have recently described that DNAm levels at two CpGs can reliably discern between pluripotent and non-pluripotent cells (Lenz et al., 2015). In this study, we followed the hypothesis that the DNAm profile of MSCs might also reflect specific modifications that are indicative for the cell type and/or the tissue of origin. Small epigenetic signatures based on site-specific analysis of DNAm in a few CpG sites might therefore be particularly appealing for the classification of MSCs.

RESULTS

Global Comparison of DNA Methylation Profiles

We compiled a well-curated dataset of publicly available DNAm profiles that were generated on the Illumina HumanMethylation BeadChip platforms: 83 DNAm profiles analyzed on 27K BeadChips were used as a training set; and 107 DNAm profiles of 450K BeadChips were used as

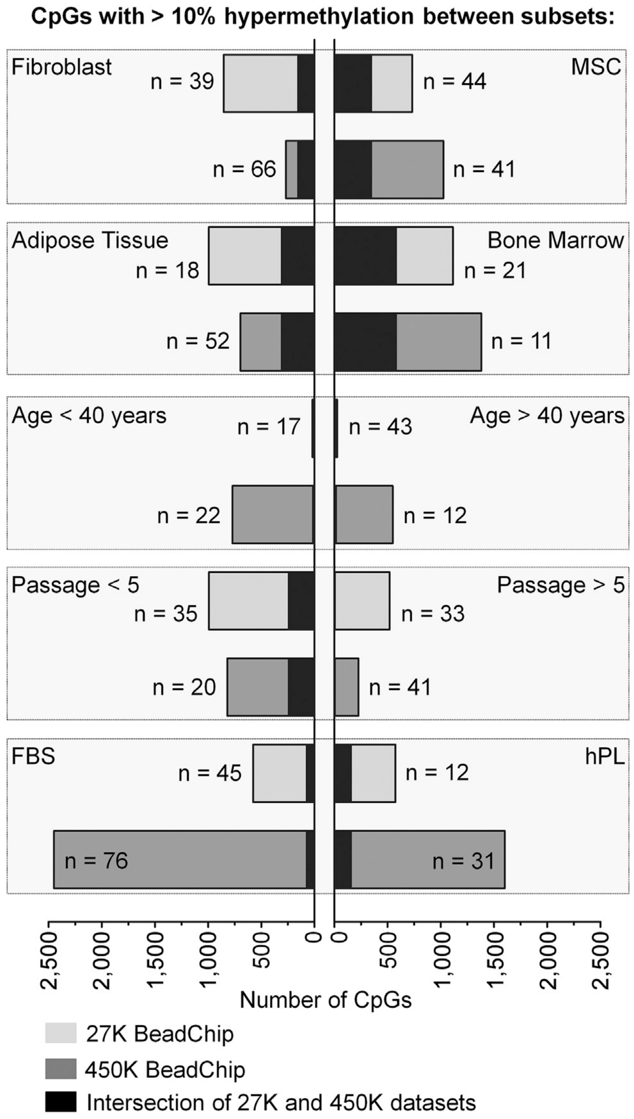

Figure 1. Differentially Methylated CpGs in Pairwise Comparisons

DNA methylation profiles (generated on Illumina HumanMethylation BeadChips 27K or 450K) were stratified by cell type (MSCs versus fibroblasts), tissue source (here particularly MSCs from bone marrow versus adipose tissue), passage (<P5 or >P5), age (<40 or >40 years), and serum supplements in culture media (human platelet lysate [hPL] versus fetal calf/bovine serum [FBS]). The number of DNAm profiles per group is indicated (n) as well as the number of significant CpGs (adjusted limma t test: $p < 0.05$ and >10% difference in mean DNAm). Overlapping CpGs in the 27K and 450K datasets are indicated by black bars.

independent validation sets (Tables S1 and S2). Therefore, we focused on 25,014 CpGs that were represented by both platforms. Initially, we performed principal-component analysis (PCA) to estimate the impact of cell type (MSCs or fibroblasts), tissue source (bone marrow [BM], adipose tissue [AT], lung, dermis, etc.), age (stratified by 40 years), passage (stratified by P5), or serum supplement (human platelet lysate [hPL] versus fetal calf/bovine serum [FBS]) on the global DNAm patterns. However, none of the major PCA components could clearly classify cell preparations according to these parameters, and there was only a moderate tendency in the comparisons: MSCs versus fibroblasts, and MSCs derived from BM versus AT (Figure S1).

Subsequently, we determined the number of differentially methylated CpGs in pairwise comparisons (adjusted limma t test: $p < 0.05$ and at least 10% differential DNAm level). This was performed independently for the 27K-BeadChip training and the 450K-BeadChip validation set. To roughly estimate the reproducibility of DNAm differences, we then focused on CpGs with overlapping DNAm changes in both datasets (Figure 1): 346 and 152 CpGs were methylated higher in MSCs and fibroblasts, respectively, indicating that there are reproducible epigenetic differences between the two cell types. Furthermore, 580 and 307 CpGs were differentially methylated in MSCs from BM versus AT. There were hardly any overlapping age-related DNAm differences in samples from younger or older donors, although it has been shown that age-related DNAm patterns persist in MSCs (Frobel et al., 2014; Weidner et al., 2014). This might be due to the classification into two age groups, whereas age-related changes are continuously acquired throughout life. In analogy, we observed only 242 CpGs that were methylated higher at early passages (<P5) compared with late passages (>P5), although many DNAm changes were shown to be continuously hyper- and hypomethylated during culture expansion (Koch et al., 2013). Serum supplements seemed to induce rather few DNAm changes. Taken together, global analysis indicated that particularly cell type and tissue of origin are reflected by specific DNAm changes.

Epigenetic Score for Classification into MSCs and Fibroblasts

To identify CpGs that facilitate the best discrimination of MSCs and fibroblasts in the 27K-BeadChip training set, we selected CpGs with (1) the highest difference in mean DNAm in MSCs versus fibroblasts, and (2) small variation in DNAm levels within each of the two cell types (Figure 2A). Only three and nine CpGs revealed more than 40% higher DNAm levels in MSCs and fibroblasts, respectively (Figure 2B). These CpGs were subsequently plotted against the sum of variances in MSCs and fibroblasts, and thereby we identified four candidate CpGs that were associated with serpin peptidase inhibitor B5 (*SERPINB5*: cg00226904), chromosome 3 open reading

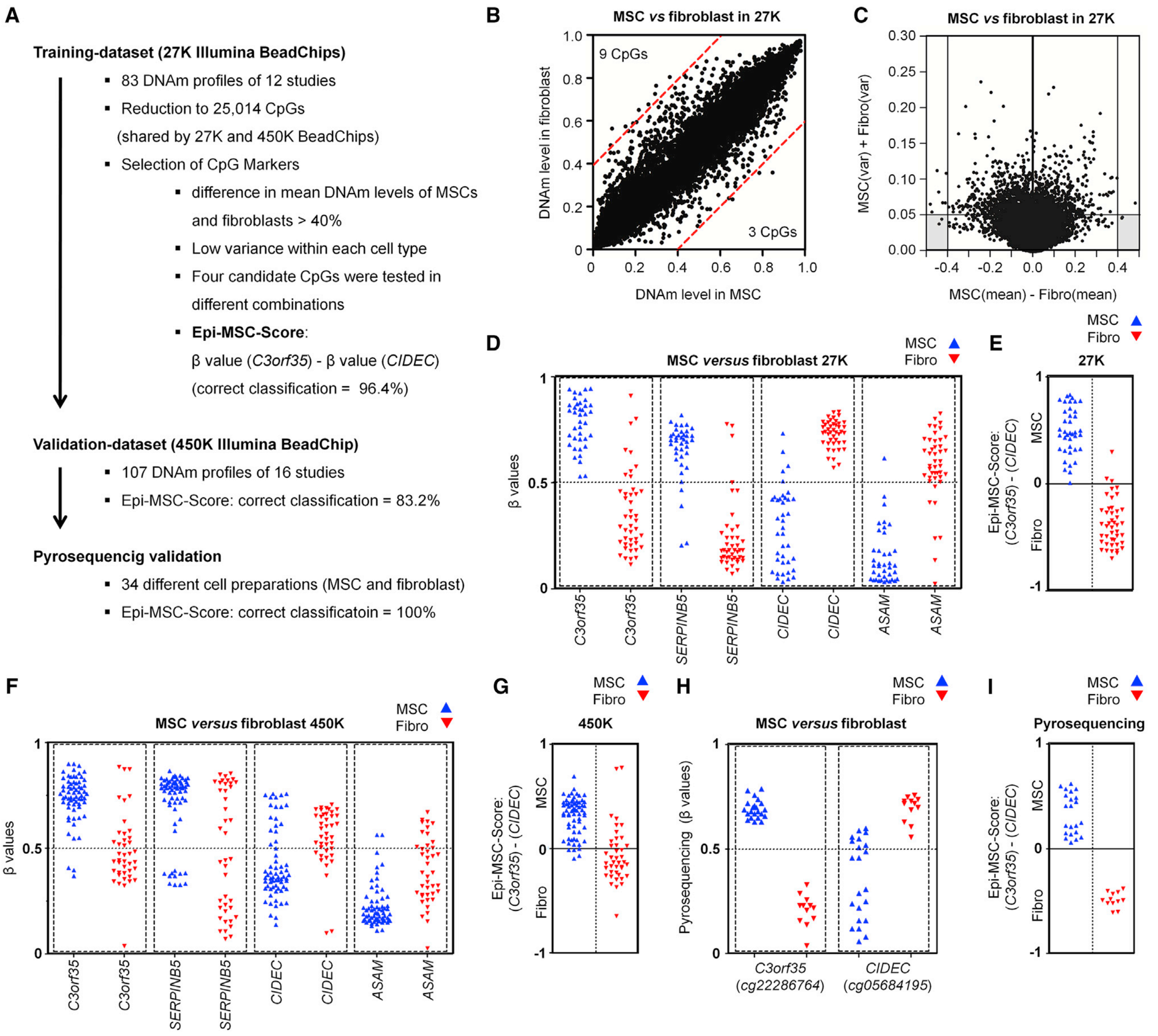

Figure 2. Epigenetic Classification of MSCs and Fibroblasts

(A) Schematic overview of the experimental design that led to the Epi-MSC-Score.

(B) Scatterplot of mean DNAm levels of MSCs and fibroblasts in the training dataset (CpGs with more than 40% difference are indicated by red lines).

(C) Differential DNAm levels were plotted against the sum of variances within MSCs and fibroblasts.

(D) DNAm levels (β values) of four CpGs that have been selected from the training datasets (27K BeadChips).

(E) Classification of the training dataset by the Epi-MSC-Score. This score represents the difference of β values at cg22286764 (*C3orf35*) and cg05684195 (*CIDEC*).

(F) DNAm levels of the four selected CpGs in the validation dataset (450K BeadChips; in analogy to Figure 2D).

(G) Classification of the validation dataset by the Epi-MSC-Score.

(H) Pyrosequencing analysis of DNAm at the two CpGs corresponding to the Epi-MSC-Score in 34 different cell preparations.

(I) Classification of pyrosequencing results by the Epi-MSC-Score based on CpG in *C3orf35* and *CIDEC* as indicated.

frame 35 (*C3orf35*: cg22286764), cell death-inducing DFFA-like effector C (*CIDEC*: cg05684195), and adipocyte-specific adhesion molecule (*ASAM*: cg19096475; Figures 2C and 2D). Iterative pair combinations of these CpGs demonstrated that the difference in DNAm at the CpGs in *C3orf35* and *CIDEC*, subsequently referred to as the

Epi-MSC-Score, could best discern MSCs from fibroblasts: a positive score is indicative of MSCs and 96% of samples were correctly classified in the 27K-BeadChip training set (Figure 2E). We repeated the analysis after resampling the training set with bootstrapping, and the two CpGs were among the top eight stable CpG sites (Supplemental Experimental Procedures). In the independent 450K-BeadChip validation set, all four candidate CpGs revealed the same trend (Figure 2F) and 83% of the samples were classified correctly (Figure 2G). Overall the differences in mean DNAm levels in MSCs versus fibroblasts were smaller in this dataset. However, applying the two aforementioned criteria for selection of relevant CpGs on the 450K dataset demonstrated that the two CpGs in *C3orf35* and *CIDEC* were again among the best performing (data not shown).

We then designed pyrosequencing assays for these two regions to facilitate robust and more quantitative analysis of the DNAm levels at the two relevant CpG sites (Figure S2A). These pyrosequencing assays were tested on 34 primary cell preparations, all of which were correctly classified into MSCs and fibroblasts (Figures 2H and 2I). Gene-expression profiles demonstrated slightly higher expression of *C3orf35* and *CIDEC* in MSCs (Figure S2B). Thus, the Epi-MSC-Score can be used for the classification of MSCs and fibroblasts.

Epigenetic Score to Discern MSCs from Bone Marrow and Adipose Tissue

We extended this analysis to derive an "Epi-Tissue-Score" for discerning MSCs that were initially isolated from either BM or AT, since these tissues are most frequently used for isolation of MSCs (Figure 3A). 29 and 30 CpGs revealed a more than 40% higher mean DNAm level in MSCs from either BM or AT, respectively (Figure 3B). We focused on 12 CpGs with lowest variances within each of these groups, which were associated with: solute carrier family 41 magnesium transporter member 2 (*SLC41A2*: cg27149093); single-minded family BHLH transcription factor 2 (*SIM2*: cg02672220); four and a half LIM domains 2 (*FHL2*: cg10635061); transmembrane 4 six family member 1 (*TM4SF1*: cg08124030); src-like-adaptor (*SLA*: cg02794695); runt-related transcription factor 1 (*RUNX1*: cg19836199); guanylate cyclase 1, soluble, beta 2 (*GUCY1B2*: cg16692277); urocortin 2 (*UCN2*: cg05125838); interleukin-26 (*IL26*: cg25697314); ecotropic viral integration site 2B (*EVI2B*: cg05109049); tubulin tyrosine ligase-like family member 3 (*TTLL3*: cg03375833); and intestinal trefoil factor 3 (*TFF3*: cg04806409; Figures 3C and 3D). The difference between the DNAm levels of the CpGs in *SLC41A2* and *TM4SF1* showed best discrimination in the 27K-BeadChip training set (100% correctly classified) and was therefore considered as the Epi-Tissue-Score (Figure 3E). Notably, all 12 candidate CpGs demonstrated tissue type-specific DNAm patterns also in the 450K-BeadChip validation set (Figure 3F), and 98.4% of these samples were correctly classified by the Epi-Tissue-Score (Figure 3G). Pyrosequencing assays were designed for the two CpGs in *SLC41A2* and *TM4SF1* (Figure S3A), and thereby 22 analyzed MSC preparations were correctly classified into BM- or AT-derived MSCs (Figures 3H and 3I). We also observed moderate differences in gene expression of *SLC41A2* and *TM4SF1* between MSCs from BM and AT (Figure S3B). Our analysis pinpoints clear molecular differences in MSCs that have been isolated from BM or AT, which can be reliably tracked by the Epi-Tissue-Score.

Epigenetic Classification of iPSC-Derived MSCs

We have recently demonstrated differentiation of induced pluripotent stem cells (iPSCs) toward MSCs, referred to as iPS-MSCs (Frobel et al., 2014). The DNAm profiles of these iPS-MSCs were now compared with those of primary cell preparations: iPS-MSCs were classified as MSCs by the Epi-MSC-Score (Figures S4A and S4B), and this was validated by pyrosequencing analysis of additional iPS-MSC preparations (Figure S4F). In contrast, the DNAm patterns at the 12 tissue-specific CpGs were not clearly indicative of BM- or AT-derived MSCs (Figure S4C). PCA analysis using either the four cell type-specific or the 12 tissue-specific CpGs supported the notion that iPS-MSCs are related to MSCs, whereas they do not reflect a clear tissue-specific association (Figures S4D and S4E). This is in line with our previous report that tissue-specific patterns are erased by reprogramming into iPSCs (Shao et al., 2013), and overall are not reestablished upon differentiation of iPSCs toward MSCs (Frobel et al., 2014).

Epigenetic Classification of Subclones

Mesenchymal stem cells comprise heterogeneous subpopulations (Cai et al., 2014; Schellenberg et al., 2012), and we have therefore challenged our epigenetic signatures on subclones. MSC cultures were seeded in 96-well plates in limiting dilutions and analyzed after 2 weeks. Additional 96-well plates were further differentiated toward adipogenic or osteogenic lineages for 2 weeks (Figure S4G). The individual subclones revealed very heterogeneous in vitro differentiation potential, as described in our previous work (Schellenberg et al., 2012), and could therefore be classified into clones with high or low differentiation potential (Figure 4A). Adipogenic differentiation potential was estimated by the percentage of cells harboring fat droplets (stained with BODIPY) and osteogenic differentiation by the amount of calcium phosphate precipitates (stained

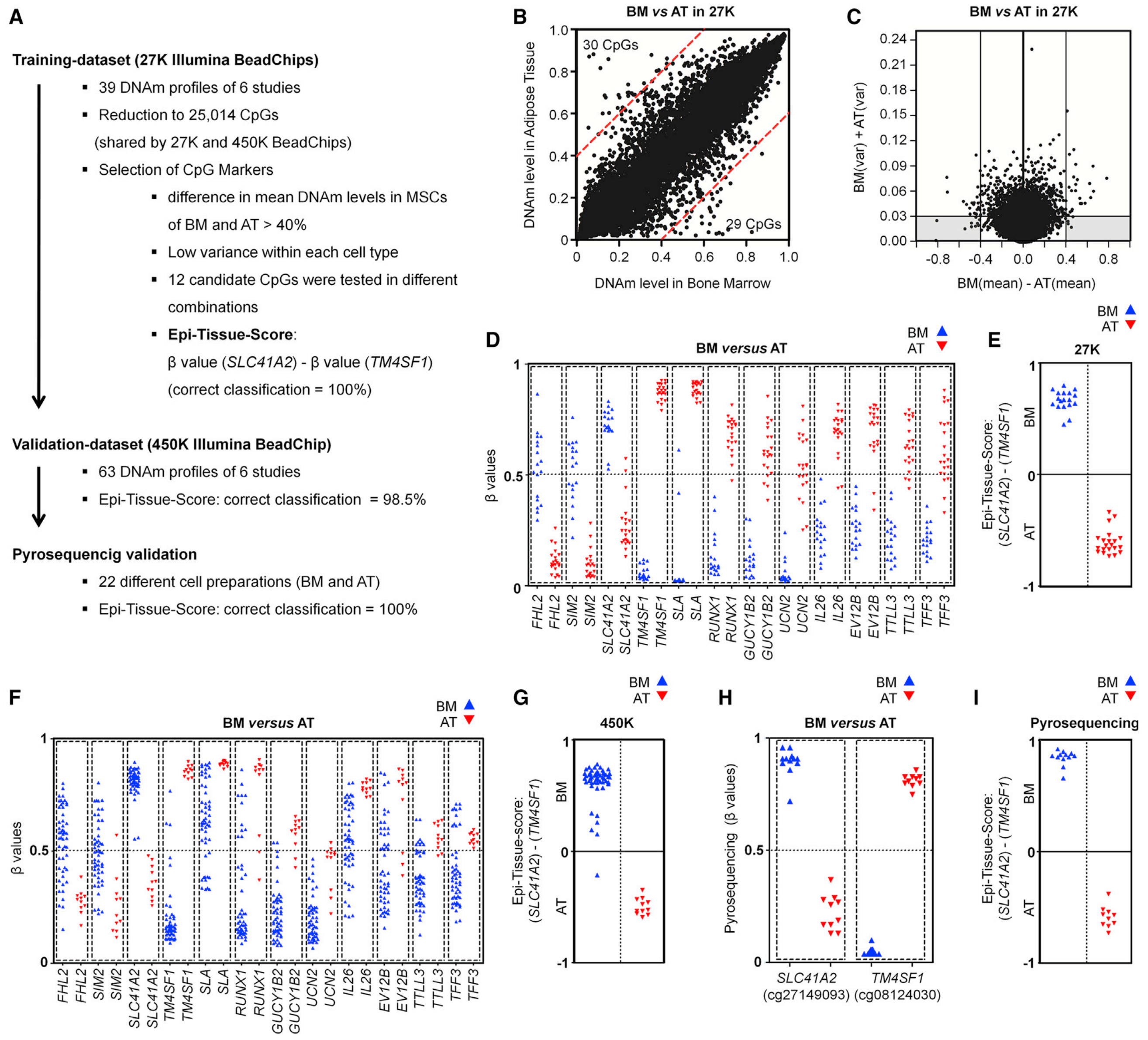

Figure 3. Classification of MSCs from Bone Marrow and Adipose Tissue

(A) Schematic overview of experimental design that led to the Epi-Tissue-Score.

(B) Scatterplot of mean DNAm levels in MSCs from bone marrow (BM) versus MSCs from adipose tissue (AT) in the training set (27K BeadChips; CpGs with more than 40% difference are indicated by red lines).

(C) Differential DNAm levels were plotted against the sum of variances within MSCs derived from either BM or AT.

(D) β Values (DNAm levels) of 12 CpGs that were selected by these criteria.

(E) Classification of the training dataset by the Epi-Tissue-Score. This score represents the difference of β values at cg27149093 (*SLC41A2*) and cg08124030 (*TM4SF1*).

(F) DNAm levels of the 12 selected CpGs in the validation dataset (450K BeadChips; in analogy to Figure 3D).

(G) Classification of the validation dataset by the Epi-Tissue-Score.

(H) Pyrosequencing analysis of DNAm at the two CpGs corresponding to the Epi-Tissue-Score in 22 MSC samples from BM and AT.

(I) Classification of pyrosequencing results by the Epi-Tissue-Score based on CpG in *SLC41A2* and *TM4SF1* as indicated.

with Alizarin red; Figure 4B). DNA of 30 clones was subsequently harvested and analyzed with our Epi-MSC-Score and Epi-Tissue-Score. All subclones were correctly classified as BM-derived MSCs, irrespective of their in vitro differentiation potential (Figures 4C, 4D, S4H, and S4I). This indicates that the epigenetic classification is not due to shifts

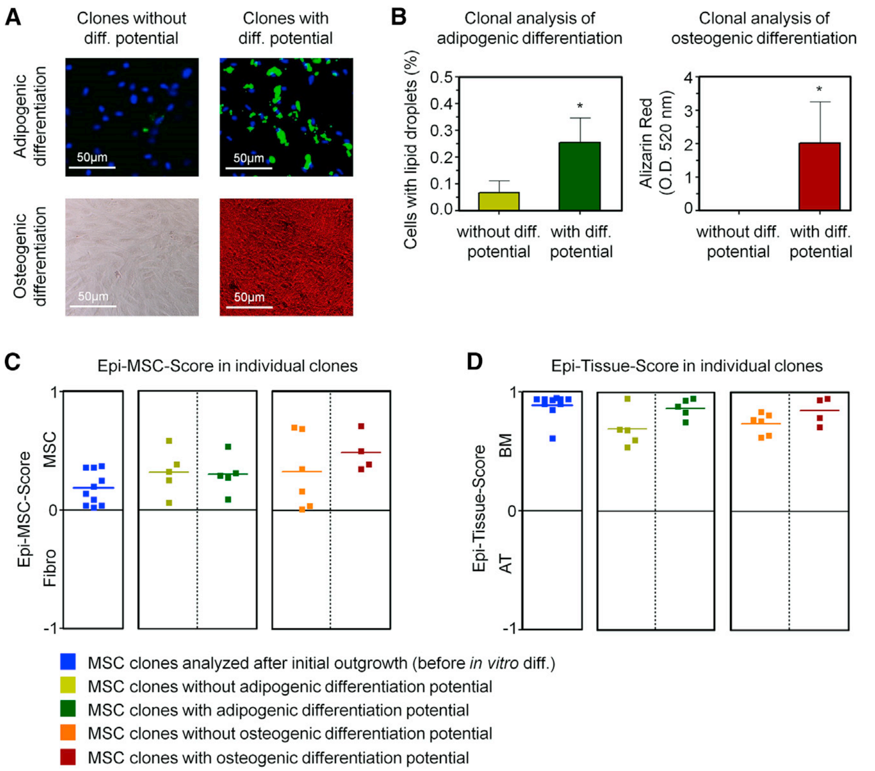

Figure 4. Analysis of Epigenetic Scores in Subclones of MSCs

(A) Bone marrow-derived MSCs were subcloned and differentiated toward adipogenic or osteogenic lineages (stained with BODIPY/DAPI or Alizarin red, respectively). Representative images of clones with low or high differentiation potential are shown.

(B) The in vitro differentiation potential toward adipogenic and osteogenic lineages was determined based on the percentage of cells with fat droplets or absorbance of Alizarin staining, respectively. For subsequent pyrosequencing analysis, we selected five clones that revealed either higher or lower differentiation (Student's t test; $*p < 0.05$; error bars represent the SD).

(C and D) Classification of MSC clones based on pyrosequencing results by Epi-MSC-Score (C) and Epi-Tissue-Score (D).

in the cellular composition, and rather reflects cell-intrinsic molecular characteristics.

DISCUSSION

Reliable measures for quality control are a prerequisite for the standardization of cell preparations to be used in experimental studies and cellular therapy. Here, we demonstrate that epigenetic signatures can support the classification of MSCs. In general, the precision of signatures can be increased by using a higher number of CpGs, but this requires more complex or even genome-wide analysis. Our two CpGs scores, which are based on one hypermethylated and one hypomethylated CpG site, are therefore a tradeoff to facilitate fast, cost-effective, and transparent classification.

Despite extensive efforts, it remains a challenge to distinguish between fibroblasts and MSCs. This definition is usually based on the in vitro differentiation potential of MSCs, although these surrogate assays hardly facilitate quantitative comparison, particularly not between different laboratories (Bortolotti et al., 2015; Dominici et al., 2006; Hematti, 2012). In our comparative study, we had to rely on the classification provided by the authors who deposited the DNAm profiles. Hence, they are not based on common standards in cell culture and quality control. At least for the cell preparations that we analyzed by pyrosequencing, we consistently observed higher differentiation potential in MSCs compared with fibroblasts (Koch et al., 2011), and these were all correctly classified by the Epi-MSC-Score. On the other hand, our clonal analysis indicated that this signature is not directly associated with the subset in MSCs that reveals higher in vitro differentiation potential.

The epigenome reflects the tissue of origin even after long-term culture (Reinisch et al., 2015; Schellenberg et al., 2012). MSCs can be isolated from a multitude of different tissues (Crisan et al., 2008), but the vast majority of studies utilize MSCs from BM and AT. In fact, cell preparations derived from other tissues are often rather referred to as fibroblasts, and therefore classification of the Epi-MSC-Score may partly be also attributed to the different tissue sources. Either way, classifications with the Epi-MSC-Score are generally in line with those provided by the corresponding publications. Furthermore, the Epi-Tissue-Score can very reliably distinguish between MSCs from BM and AT. The remarkable difference in the epigenetic makeup of MSCs from different tissues, which are cell intrinsic and not due to cellular heterogeneity, may reflect the stark tissue-specific differences in gene-expression profiles (Wagner et al., 2005), proteome (Wagner et al., 2006), and functional readouts (Reinisch et al., 2015). All the more, such analysis is relevant for quality control.

Researchers are usually aware of the tissue that was initially used for isolation of MSCs, but there is evidence that accidental interchange of samples or contaminations with other cells can occur (Garcia et al., 2010; Torsvik et al., 2010). For established cell lines, some contaminations can be detected by specific SNPs or mutations, but for primary cells with unknown genetic background this can hardly be unraveled. In this regard, our epigenetic signatures provide a perspective for quality control of cell preparations. We expect that the signatures can be further fine-tuned based on the rapidly growing number of available DNAm datasets. This will also facilitate generation of other epigenetic signatures reflecting functional properties of MSCs, such as their immunomodulatory potential or the hematopoiesis supportive function (Wuchter et al., 2015). It is even conceivable that epigenetic signatures can be developed to estimate the therapeutic potential of MSCs, but such predictors need to be specifically trained and validated on suitable datasets. In this regard, our exploratory study provides an alternative concept for the definition, characterization, and classification of MSCs.

EXPERIMENTAL PROCEDURES

A detailed description of all Experimental Procedures used is presented in Supplemental Experimental Procedures.

DNA Methylation Datasets

Illumina Human Methylation BeadChip datasets (27K or 450K) of MSCs and fibroblasts were retrieved from the NCBI Gene Expression Omnibus (Tables S1 and S2).

Derivation of Epigenetic Scores

To identify the best suited biomarkers for classification, we selected CpG sites with high differences in mean DNAm levels (>40% of difference) and low variance within groups. A hypermethylated and a hypomethylated CpG were then utilized for each score as follows: Epi-MSC-Score = β value at cg22286764 (*C3orf35*) minus the β value at cg05684195 (*CIDEC*); and Epi-Tissue-Score = β value at cg27149093 (*SLC41A2*) minus the β value at cg08124030 (*TM4SF1*). Both scores range from -1 to 1; positive values indicate MSCs and BM, and negative ones fibroblast and AT, respectively.

Primary Cells

All cells were taken after written consent was granted, and have been specifically approved by the local Ethics Committees for Use of Human Subjects at RWTH Aachen University (permit numbers: BM-MSC: *#EK128/09*; AT-MSCs: *#EK187/08*; fibroblasts: *#EK187/08*). Cell culture, immunophenotyping, and in vitro differentiation were performed as described previously (Frobel et al., 2014; Koch et al., 2011). Additional Information about the samples is provided in Table S3. For clonal analysis, MSCs at passage 1–2 (n = 3) were submitted to the limiting dilutions in 96-well plates of 1, 3, 10, and 30 cells per well as described previously (Schellenberg et al., 2012).

Pyrosequencing Analysis

Genomic DNA was isolated from 10^6 cells (bulk culture) or clones in 96-well plates using the NucleoSpin Tissue/Tissue XS kits (Macherey-Nagel) and quantified with an ND-1000 spectrometer (NanoDrop). Between 100 and 1,000 ng of DNA was sodium bisulfite-converted using the EZ DNA Methylation kit (Zymo Research), and PCR procedures and sequencing assays were performed using the PyroMark PCR and Q96 kits (Qiagen) (Lenz et al., 2015). Primers are specified in Table S4.

SUPPLEMENTAL INFORMATION

Supplemental Information includes Supplemental Experimental Procedures, four figures, and four tables and can be found with this article online at http://dx.doi.org/10.1016/j.stemcr.2016.01.003.

ACKNOWLEDGMENTS

This work was supported by the Else Kröner-Fresenius Stiftung (2014_A193), the German Ministry of Education and Research (BMBF; OBELICS), and the Interdisciplinary Center for Clinical Research (IZKF) in the Faculty of Medicine at the RWTH Aachen University (T11-2). Wolfgang Wagner is cofounder of Cygenia GmbH (www.cygenia.com), which may provide service for the Epi-MSC-Score and the Epi-Tissue-Score to other scientists.

Received: November 2, 2015
Revised: January 4, 2016
Accepted: January 7, 2016
Published: February 9, 2016

REFERENCES

Bae, S., Ahn, J.H., Park, C.W., Son, H.K., Kim, K.S., Lim, N.K., Jeon, C.J., and Kim, H. (2009). Gene and microRNA expression signatures of human mesenchymal stromal cells in comparison to fibroblasts. Cell Tissue Res. *335*, 565–573.

Bortolotti, F., Ukovich, L., Razban, V., Martinelli, V., Ruozi, G., Pelos, B., Dore, F., Giacca, M., and Zacchigna, S. (2015). In vivo therapeutic potential of mesenchymal stromal cells depends on the source and the isolation procedure. Stem Cell Rep. *4*, 332–339.

Buhring, H.J., Battula, V.L., Treml, S., Schewe, B., Kanz, L., and Vogel, W. (2007). Novel markers for the prospective isolation of human MSC. Ann. N. Y. Acad. Sci. *1106*, 262–271.

Cai, J., Miao, X., Li, Y., Smith, C., Tsang, K., Cheng, L., and Wang, Q.F. (2014). Whole-genome sequencing identifies genetic variances in culture-expanded human mesenchymal stem cells. Stem Cell Rep. *3*, 227–233.

Crisan, M., Yap, S., Casteilla, L., Chen, C.W., Corselli, M., Park, T.S., Andriolo, G., Sun, B., Zheng, B., Zhang, L., et al. (2008). A perivascular origin for mesenchymal stem cells in multiple human organs. Cell Stem Cell *3*, 301–313.

Dominici, M., Le Blanc, K., Mueller, I., Slaper-Cortenbach, I., Marini, F., Krause, D., Deans, R., Keating, A., Prockop, D., and Horwitz, E. (2006). Minimal criteria for defining multipotent mesenchymal

stromal cells. The International Society for Cellular Therapy position statement. Cytotherapy *8*, 315–317.

Erdmann, G., Suchanek, M., Horn, P., Graf, F., Volz, C., Horn, T., Zhang, X., Wagner, W., Ho, A.D., and Boutros, M. (2015). Functional fingerprinting of human mesenchymal stem cells using high-throughput RNAi screening. Genome Med. *7*, 46.

Frobel, J., Hemeda, H., Lenz, M., Abagnale, G., Joussen, S., Denecke, B., Saric, T., Zenke, M., and Wagner, W. (2014). Epigenetic rejuvenation of mesenchymal stromal cells derived from induced pluripotent stem cells. Stem Cell Rep. *3*, 414–422.

Garcia, S., Bernad, A., Martin, M.C., Cigudosa, J.C., Garcia-Castro, J., and de la Fuente, R. (2010). Pitfalls in spontaneous in vitro transformation of human mesenchymal stem cells. Exp. Cell Res. *316*, 1648–1650.

Halfon, S., Abramov, N., Grinblat, B., and Ginis, I. (2011). Markers distinguishing mesenchymal stem cells from fibroblasts are downregulated with passaging. Stem Cells Dev. *20*, 53–66.

Hematti, P. (2012). Mesenchymal stromal cells and fibroblasts: a case of mistaken identity? Cytotherapy *14*, 516–521.

Holley, R.J., Tai, G., Williamson, A.J., Taylor, S., Cain, S.A., Richardson, S.M., Merry, C.L., Whetton, A.D., Kielty, C.M., and Canfield, A.E. (2015). Comparative quantification of the surfaceome of human multipotent mesenchymal progenitor cells. Stem Cell Rep. *4*, 473–488.

Ishii, M., Koike, C., Igarashi, A., Yamanaka, K., Pan, H., Higashi, Y., Kawaguchi, H., Sugiyama, M., Kamata, N., Iwata, T., et al. (2005). Molecular markers distinguish bone marrow mesenchymal stem cells from fibroblasts. Biochem. Biophys. Res. Commun. *332*, 297–303.

Jaenisch, R., and Bird, A. (2003). Epigenetic regulation of gene expression: how the genome integrates intrinsic and environmental signals. Nat. Genet. Suppl. *33*, 245–254.

Karnik, R., and Meissner, A. (2013). Browsing (Epi)genomes: a guide to data resources and epigenome browsers for stem cell researchers. Cell Stem Cell *13*, 14–21.

Koch, C.M., Suschek, C.V., Lin, Q., Bork, S., Goergens, M., Joussen, S., Pallua, N., Ho, A.D., Zenke, M., and Wagner, W. (2011). Specific age-associated DNA methylation changes in human dermal fibroblasts. PLoS One *6*, e16679.

Koch, C.M., Reck, K., Shao, K., Lin, Q., Joussen, S., Ziegler, P., Walenda, G., Drescher, W., Opalka, B., May, T., et al. (2013). Pluripotent stem cells escape from senescence-associated DNA methylation changes. Genome Res. *23*, 248–259.

Lenz, M., Goetzke, R., Schenk, A., Schubert, C., Veeck, J., Hemeda, H., Koschmieder, S., Zenke, M., Schuppert, A., and Wagner, W. (2015). Epigenetic biomarker to support classification into pluripotent and non-pluripotent cells. Sci. Rep. *5*, 8973.

Reinisch, A., Etchart, N., Thomas, D., Hofmann, N.A., Fruehwirth, M., Sinha, S., Chan, C.K., Senarath-Yapa, K., Seo, E.Y., Wearda, T., et al. (2015). Epigenetic and in vivo comparison of diverse MSC sources reveals an endochondral signature for human hematopoietic niche formation. Blood *125*, 249–260.

Schellenberg, A., Stiehl, T., Horn, P., Joussen, S., Pallua, N., Ho, A.D., and Wagner, W. (2012). Population dynamics of mesenchymal stromal cells during culture expansion. Cytotherapy *14*, 401–411.

Shao, K., Koch, C., Gupta, M.K., Lin, Q., Lenz, M., Laufs, S., Denecke, B., Schmidt, M., Linke, M., Hennies, H.C., et al. (2013). Induced pluripotent mesenchymal stromal cell clones retain donor-derived differences in DNA methylation profiles. Mol. Ther. *21*, 240–250.

Sorrentino, A., Ferracin, M., Castelli, G., Biffoni, M., Tomaselli, G., Baiocchi, M., Fatica, A., Negrini, M., Peschle, C., and Valtieri, M. (2008). Isolation and characterization of CD146+ multipotent mesenchymal stromal cells. Exp. Hematol. *36*, 1035–1046.

Squillaro, T., Peluso, G., and Galderisi, U. (2015). Clinical trials with mesenchymal stem cells: an update. Cell Transplant. http://dx.doi.org/10.3727/096368915X689622.

Torsvik, A., Rosland, G.V., Svendsen, A., Molven, A., Immervoll, H., McCormack, E., Lonning, P.E., Primon, M., Sobala, E., Tonn, J.C., et al. (2010). Spontaneous malignant transformation of human mesenchymal stem cells reflects cross-contamination: putting the research field on track—letter. Cancer Res. *70*, 6393–6396.

Wagner, W., Wein, F., Seckinger, A., Frankhauser, M., Wirkner, U., Krause, U., Blake, J., Schwager, C., Eckstein, V., Ansorge, W., et al. (2005). Comparative characteristics of mesenchymal stem cells from human bone marrow, adipose tissue, and umbilical cord blood. Exp. Hematol. *33*, 1402–1416.

Wagner, W., Feldmann, R.E., Jr., Seckinger, A., Maurer, M.H., Wein, F., Blake, J., Krause, U., Kalenka, A., Burgers, H.F., Saffrich, R., et al. (2006). The heterogeneity of human mesenchymal stem cell preparations—evidence from simultaneous analysis of proteomes and transcriptomes. Exp. Hematol. *34*, 536–548.

Weidner, C.I., Lin, Q., Koch, C.M., Eisele, L., Beier, F., Ziegler, P., Bauerschlag, D.O., Jockel, K.H., Erbel, R., Muhleisen, T.W., et al. (2014). Aging of blood can be tracked by DNA methylation changes at just three CpG sites. Genome Biol. *15*, R24.

Wuchter, P., Bieback, K., Schrezenmeier, H., Bornhauser, M., Muller, L.P., Bonig, H., Wagner, W., Meisel, R., Pavel, P., Tonn, T., et al. (2015). Standardization of good manufacturing practice-compliant production of bone marrow-derived human mesenchymal stromal cells for immunotherapeutic applications. Cytotherapy *17*, 128–139.

Stem Cell Reports, Volume *6*

Supplemental Information

Epigenetic Classification of Human Mesenchymal Stromal Cells

Danilo Candido de Almeida, Marcelo R.P. Ferreira, Julia Franzen, Carola I. Weidner, Joana Frobel, Martin Zenke, Ivan G. Costa, and Wolfgang Wagner

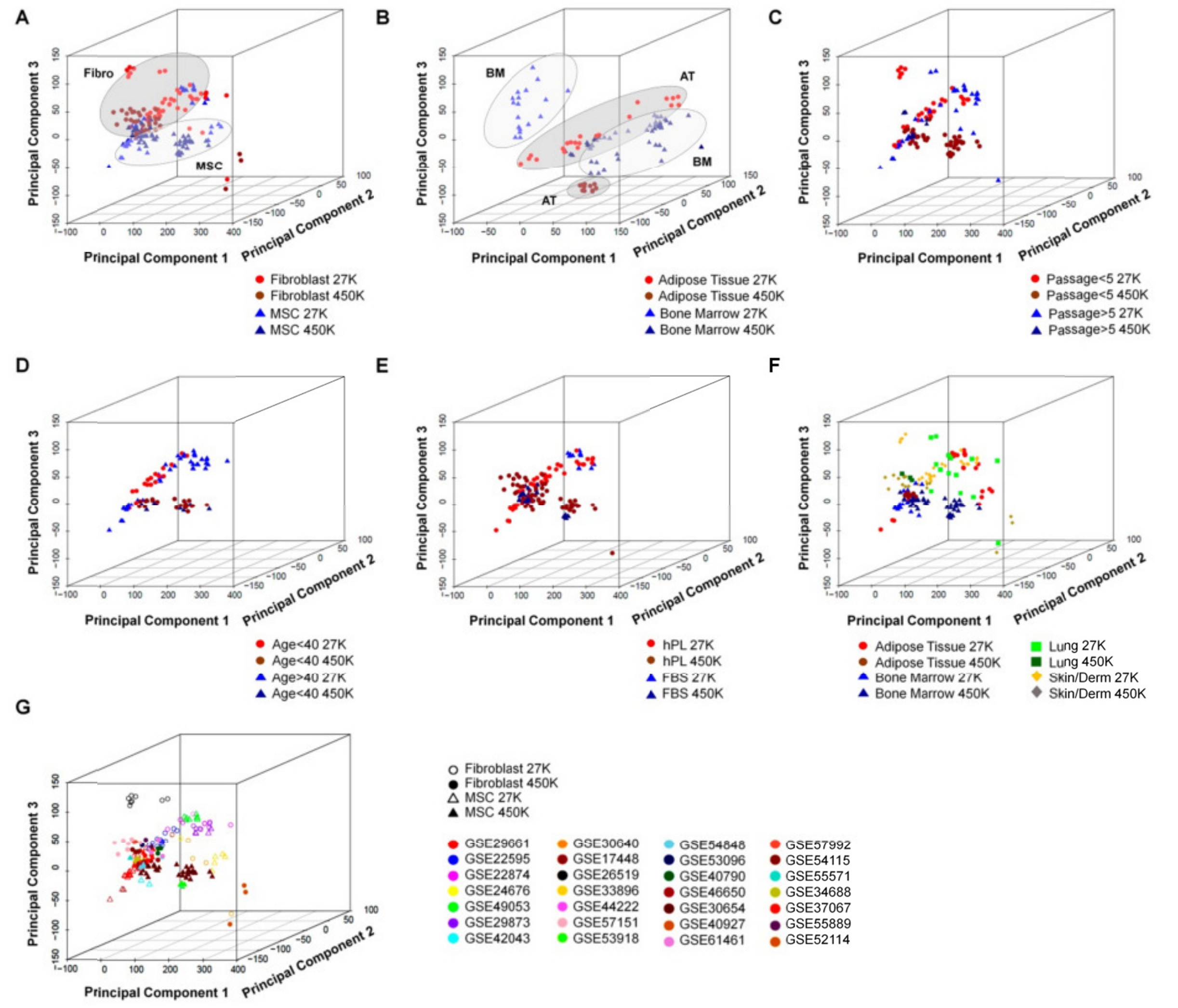

Figure S1. Principal component analysis of DNAm patterns, related to Figure 1.
Principal component analysis (PCA) of DNAm profiles according to:
(A) cell type (MSCs *versus* Fibroblasts),
(B) tissue source (MSCs from bone marrow [BM] or adipose tissue [AT]),
(C) passage (less or more than 5 passages),
(D) age (less or more than 40 years),
(E) serum supplement utilized in culture (human platelet lysate [hPL] or fetal bovine serum [FBS]), and
(F) different tissue sources,
(G) different studies (GEO IDs of the corresponding datasets are indicated)
Overall, none of these cellular characteristics was clearly reflected by major components in PCA. A moderate association was only observed for cell type and comparison of MSCs from BM and AT (indicated by shaded circular areas). Samples of individual studies clustered often together, but this may not only be due to technical reasons or normalization regimen, but rather to the different cell preparations and culture conditions used in these individual studies.

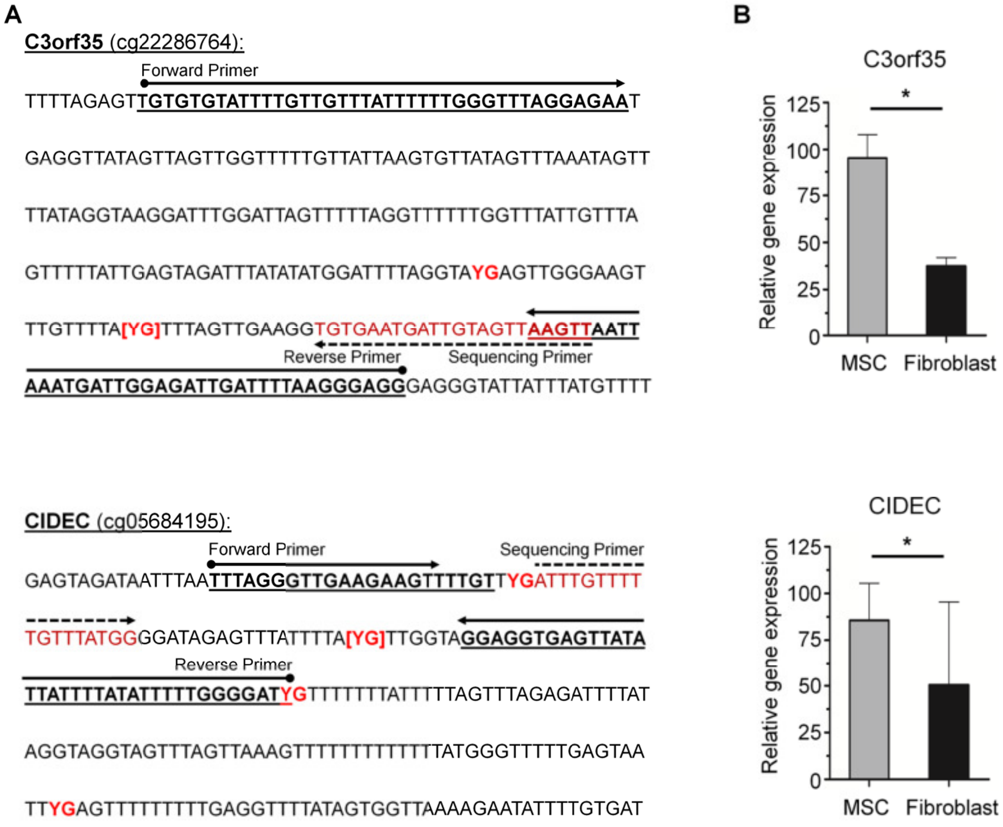

Figure S2. Pyrosequencing and gene expression for Epi-MSC-Score, related to Figure 2.
(A) Pyrosequencing assays for the two relevant CpG-sites in the Epi-MSC-Score. The genomic sequence of bisulfite-converted DNA is demonstrated. Please note that all non-methylated cytosines have been converted to thymidines; CG base pairs are therefore indicated as YGs. The relevant CpG-sites (cg22286764 and cg05684195) are highlighted in red brackets.
(B) Gene expression levels of *C3orf35* and *CIDEC* (associated with CpGs of the Epi-MSC-Score). The gene expression profiles were downloaded from GEO (all Illumina HumanHT-12 v4 platform; n = 46). Data were quantile normalized and relative gene expression levels are indicated. * $p < 0.05$ (Student's t-test).

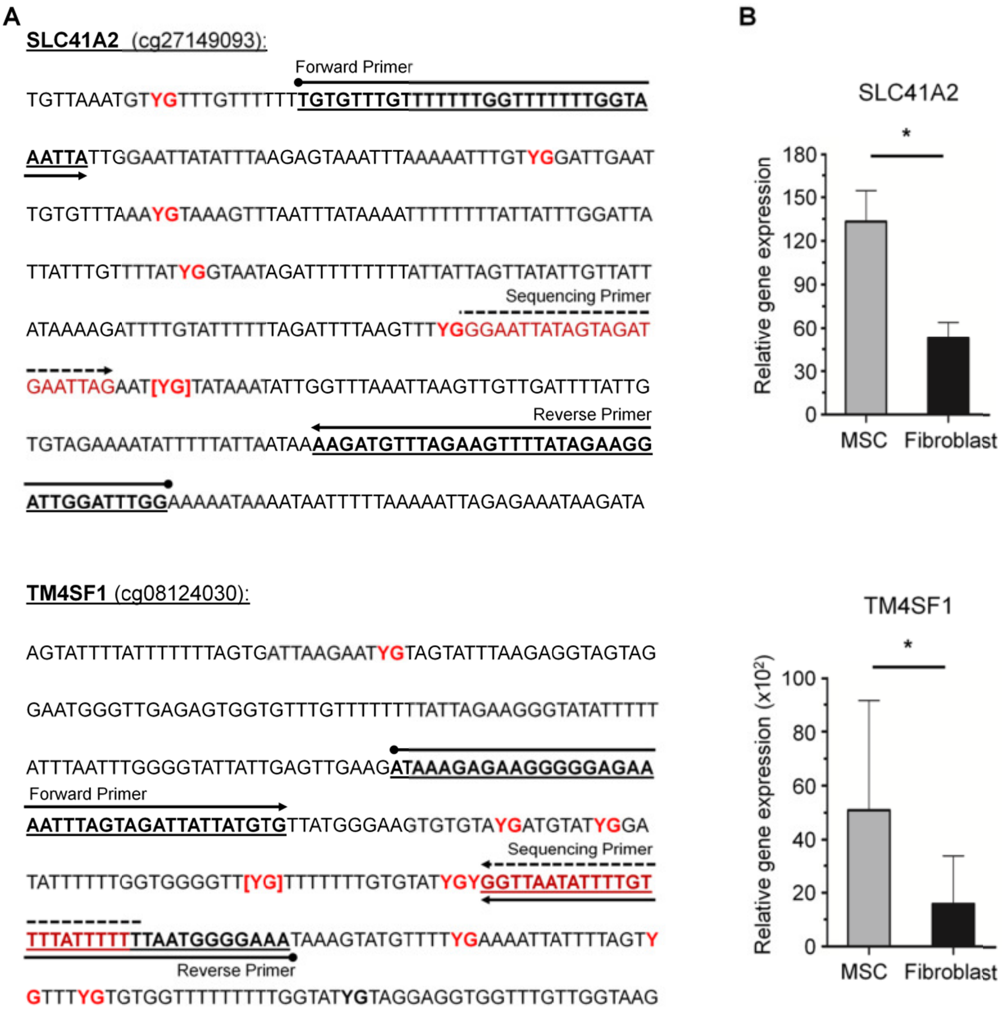

Figure S3. Pyrosequencing and gene expression for Epi-Tissue-Score, related to Figure 3.
(A) Pyrosequencing assays for the two relevant CpG-sites in the Epi-Tissue-Score. The genomic sequence of bisulfite-converted DNA is demonstrated. Please note that all non-methylated cytosines have been converted to thymidines; CG base pairs are therefore indicated as YGs. The relevant CpG-sites (cg27149093 and cg08124030) are highlighted in red brackets.
(B) Gene expression levels of *SLC41A2* and *TM4SF1* (associated with CpGs of the Epi-Tissue-Score). The gene expression profiles were downloaded from GEO (all Illumina HumanHT-12 v4 platform; n = 24). Data were quantile normalized and relative gene expression levels are indicated. * $p < 0.05$ (Student's t-test).

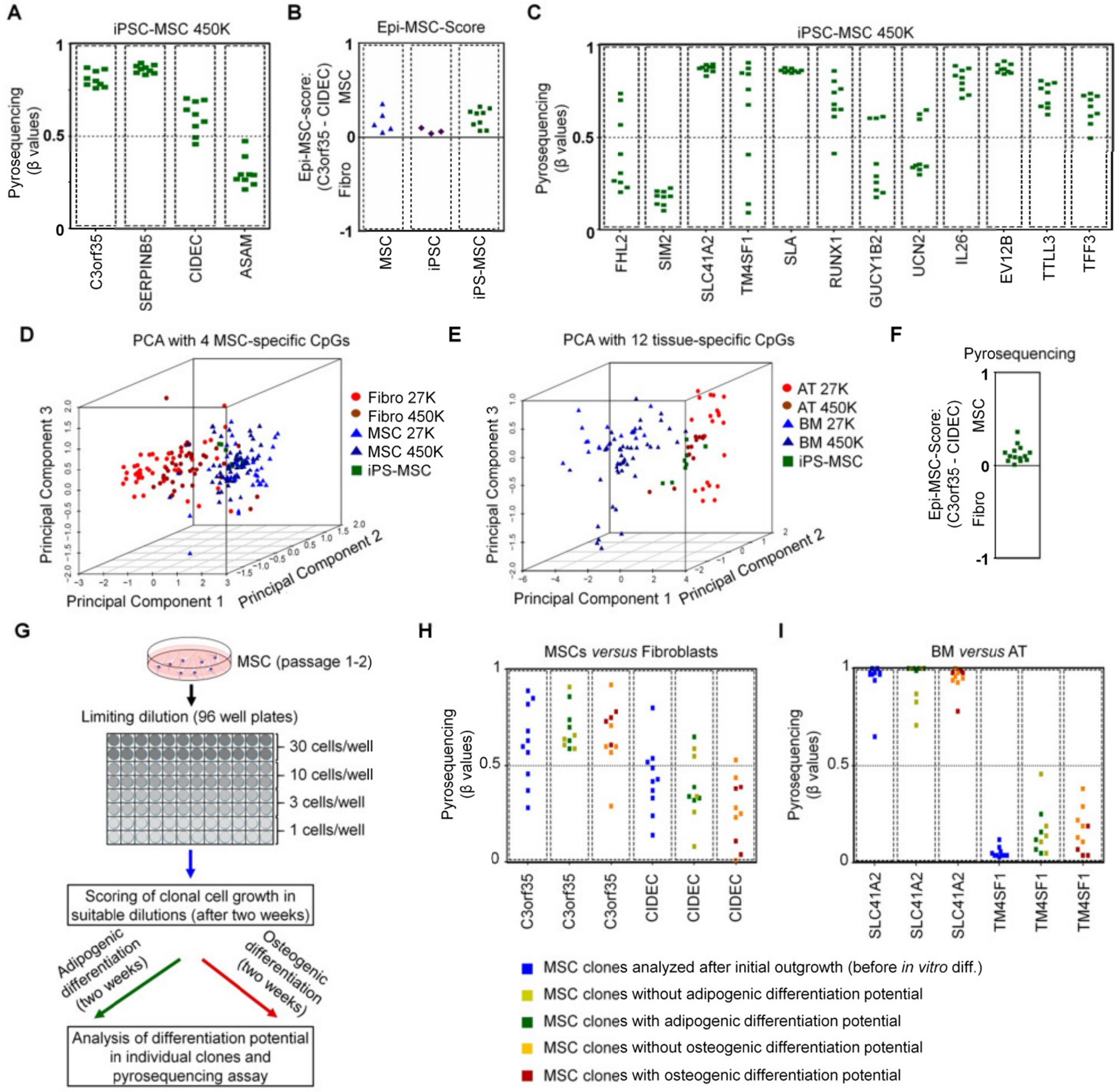

Figure S4. Classification of iPS-MSCs and of MSC clones, related to Figure 2, 3 and 4.

(A) DNAm levels of iPS-MSCs at four CpGs that can discern MSCs and fibroblasts. DNAm levels at the CpGs in *C3orf35*, *SERPINB5* and *ASAM* are overall similar to those in MSCs.

(B) The Epi-MSC-Score was used to classify parental MSCs that have been used for reprogramming, iPSCs, and iPS-MSCs. iPS-MSCs are rather classified as MSCs than as fibroblasts.

(C) DNAm levels of iPS-MSCs at 12 CpG-sites that are indicative for MSCs from bone marrow (BM) or adipose tissue (AT). This pattern did not clearly reflect bone marrow origin of iPS-MSCs.

(D) Principal component analysis (PCA) of the four MSC/fibroblast-associated CpGs (indicated in A) can clearly discern MSCs and fibroblasts. The results supported the notion that iPS-MSCs are related to MSCs.

(E) PCA of the 12 tissue associated CpGs (indicated in B) can clearly discern MSCs from bone marrow and adipose tissue. iPS-MSCs were not clearly associated to either BM- or AT-derived MSCs.

(F) Epi-MSC-Score analysis of iPS-MSCs by pyrosequencing demonstrated reproducible classification of independent clones as MSCs.

(G) Schematic representation of limiting dilutions of bone marrow MSCs and their *in vitro* differentiation. Dilutions were performed for three biological replica and only those dilutions were further considered that statistically correspond to single-cell derived colonies (Schellenberg et al., 2012). Each dilution was performed in triplicate to be (i) analyzed after two weeks without differentiation (blue), (ii) further differentiated for additional two weeks towards adipogenic lineage (green), and (iii) further differentiated towards osteogenic lineage (red). The cell clones (and differentiation potential) were scored by microscopy and absorbance measurement and DNA was then harvested for pyrosequencing analysis of the epigenetic scores.

(H) Pyrosequencing results of DNAm levels at the two CpGs of the Epi-MSC-Score.

(I) Pyrosequencing results of DNAm levels at the two CpGs of the Epi-Tissue-Score.

Table S1. DNAm profiles of the training dataset (27K BeadChip)

GEO-number	Cell-Type	Source	Location	Gender	Age	Passage	Serum	References
**GSE17448**	MSC	BM	IC	-	>40	P<5	FBS	(Bork et al., 2010)
**GSE17448**	MSC	BM	CF	-	>40	P<5	FBS	(Bork et al., 2010)
**GSE17448**	MSC	BM	CF	-	>40	P<5	FBS	(Bork et al., 2010)
**GSE17448**	MSC	BM	CF	-	>40	P<5	FBS	(Bork et al., 2010)
**GSE17448**	MSC	BM	CF	-	>40	P<5	FBS	(Bork et al., 2010)
**GSE17448**	MSC	BM	CF	-	>40	P>5	FBS	(Bork et al., 2010)
**GSE17448**	MSC	BM	IC	-	>40	P>5	FBS	(Bork et al., 2010)
**GSE17448**	MSC	BM	CF	-	>40	P>5	FBS	(Bork et al., 2010)
**GSE17448**	MSC	BM	CF	-	>40	P>5	FBS	(Bork et al., 2010)
**GSE17448**	MSC	BM	CF	-	>40	P>5	FBS	(Bork et al., 2010)
**GSE17448**	MSC	BM	IC	-	<40	P<5	FBS	(Bork et al., 2010)
**GSE17448**	MSC	BM	IC	-	<40	P<5	FBS	(Bork et al., 2010)
**GSE17448**	MSC	BM	IC	-	<40	P<5	FBS	(Bork et al., 2010)
**GSE17448**	MSC	BM	IC	-	<40	P>5	FBS	(Bork et al., 2010)
**GSE17448**	MSC	BM	IC	-	<40	P>5	FBS	(Bork et al., 2010)
**GSE17448**	MSC	BM	IC	-	<40	P>5	FBS	(Bork et al., 2010)
**GSE26519**	MSC	AT	-	F	<40	P<5	hPL	(Schellenberg et al., 2011)
**GSE26519**	MSC	AT	-	F	>40	P>5	hPL	(Schellenberg et al., 2011)
**GSE26519**	MSC	AT	-	M	>40	P>5	hPL	(Schellenberg et al., 2011)
**GSE26519**	MSC	AT	-	F	>40	P>5	hPL	(Schellenberg et al., 2011)
**GSE26519**	MSC	AT	-	F	<40	P>5	hPL	(Schellenberg et al., 2011)
**GSE26519**	MSC	AT	-	F	>40	P>5	hPL	(Schellenberg et al., 2011)
**GSE26519**	MSC	AT	-	F	>40	P>5	hPL	(Schellenberg et al., 2011)
**GSE26519**	MSC	AT	-	F	>40	P>5	hPL	(Schellenberg et al., 2011)
**GSE29661**	MSC	AT	Breast	F	>40	P<5	hPL	(Koch et al., 2012)
**GSE29661**	MSC	AT	Leg	F	>40	P<5	hPL	(Koch et al., 2012)
**GSE29661**	MSC	AT	Leg	F	>40	P>5	hPL	(Koch et al., 2012)
**GSE29661**	MSC	AT	Breast	F	>40	P>5	hPL	(Koch et al., 2012)
**GSE29873**	MSC	BM	-	-	-	-	-	(Ohm et al., 2010)
**GSE29873**	MSC	BM	-	-	-	-	-	(Ohm et al., 2010)
**GSE33896**	MSC	AT	-	-	>40	P>5	FBS	(Berdasco et al., 2012)
**GSE33896**	MSC	AT	-	-	>40	P>5	FBS	(Berdasco et al., 2012)
**GSE33896**	MSC	AT	-	-	>40	P>5	FBS	(Berdasco et al., 2012)
**GSE33896**	MSC	AT	-	-	>40	P>5	FBS	(Berdasco et al., 2012)
**GSE44222**	MSC	AT	-	-	-	-	-	(Sempere et al., 2014)
**GSE44222**	MSC	AT	-	-	-	-	-	(Sempere et al., 2014)
**GSE44222**	MSC	AT	-	-	-	-	-	(Sempere et al., 2014)
**GSE44222**	MSC	AT	-	-	-	-	-	(Sempere et al., 2014)
**GSE44222**	MSC	AT	-	-	-	-	-	(Sempere et al., 2014)
**GSE22595**	Fibroblast	Derm	Skin	F	>40	P<5	FBS	(Koch et al., 2011)
**GSE22595**	Fibroblast	Derm	Skin	F	>40	P<5	FBS	(Koch et al., 2011)
**GSE22595**	Fibroblast	Derm	Skin	F	>40	P<5	FBS	(Koch et al., 2011)
**GSE22595**	Fibroblast	Derm	Skin	F	>40	P<5	FBS	(Koch et al., 2011)
**GSE22595**	Fibroblast	Derm	Skin	F	>40	P<5	FBS	(Koch et al., 2011)
**GSE22595**	Fibroblast	Derm	Skin	F	>40	P<5	FBS	(Koch et al., 2011)
**GSE22595**	Fibroblast	Derm	Skin	F	<40	P<5	FBS	(Koch et al., 2011)
**GSE22595**	Fibroblast	Derm	Skin	F	<40	P<5	FBS	(Koch et al., 2011)
**GSE22595**	Fibroblast	Derm	Skin	F	<40	P<5	FBS	(Koch et al., 2011)
**GSE22595**	Fibroblast	Derm	Skin	F	<40	P<5	FBS	(Koch et al., 2011)
**GSE22595**	Fibroblast	Derm	Skin	F	<40	P<5	FBS	(Koch et al., 2011)
**GSE22595**	Fibroblast	Derm	Skin	F	<40	P<5	FBS	(Koch et al., 2011)
**GSE22595**	Fibroblast	Derm	Skin	F	<40	P<5	FBS	(Koch et al., 2011)
**GSE22595**	Fibroblast	Derm	Skin	F	<40	P<5	FBS	(Koch et al., 2011)
**GSE22595**	Fibroblast	Derm	Skin	F	<40	P>5	FBS	(Koch et al., 2011)
**GSE22874**	Fibroblast	Lung	-	F	>40	-	-	(Navab et al., 2011)

**GSE22874**	Fibroblast	Lung	-	M	>40	-	-	(Navab et al., 2011)
**GSE22874**	Fibroblast	Lung	-	M	>40	-	-	(Navab et al., 2011)
**GSE22874**	Fibroblast	Lung	-	F	>40	-	-	(Navab et al., 2011)
**GSE22874**	Fibroblast	Lung	-	M	>40	-	-	(Navab et al., 2011)
**GSE24676**	Fibroblast	Lung	-	M	-	-	FBS	(Nishino et al., 2011)
**GSE29661**	Fibroblast	Derm	Skin	F	>40	P<5	FBS	(Koch et al., 2012)
**GSE29661**	Fibroblast	Derm	Skin	F	>40	P>5	FBS	(Koch et al., 2012)
**GSE29661**	Fibroblast	Derm	Skin	F	>40	P<5	FBS	(Koch et al., 2012)
**GSE29661**	Fibroblast	Derm	Skin	F	>40	P<5	FBS	(Koch et al., 2012)
**GSE29661**	Fibroblast	Derm	Skin	F	>40	P<5	FBS	(Koch et al., 2012)
**GSE29661**	Fibroblast	Derm	Skin	F	>40	P>5	FBS	(Koch et al., 2012)
**GSE29661**	Fibroblast	Derm	Skin	F	>40	P>5	FBS	(Koch et al., 2012)
**GSE29661**	Fibroblast	Derm	Skin	F	>40	P>5	FBS	(Koch et al., 2012)
**GSE29873**	Fibroblast	Lung	-	-	-	-	-	(Ohm et al., 2010)
**GSE30640**	Fibroblast	Lung	-	-	-	P>5	-	-
**GSE30640**	Fibroblast	Lung	-	-	-	P>5	-	-
**GSE30640**	Fibroblast	Lung	-	-	-	P>5	-	-
**GSE30640**	Fibroblast	Lung	-	-	-	P>5	-	-
**GSE30640**	Fibroblast	Lung	-	-	-	P>5	-	-
**GSE42043**	Fibroblast	Skin	-	M	-	P<5	-	(Huang et al., 2014)
**GSE42043**	Fibroblast	Skin	-	M	-	P<5	-	(Huang et al., 2014)
**GSE42043**	Fibroblast	Lung	-	F	-	P>5	-	(Huang et al., 2014)
**GSE42043**	Fibroblast	Lung	-	F	-	P>5	-	(Huang et al., 2014)
**GSE42043**	Fibroblast	-	-	F	-	P<5	-	(Huang et al., 2014)
**GSE42043**	Fibroblast	Skin	-	M	-	P<5	-	(Huang et al., 2014)
**GSE42043**	Fibroblast	-	-	M	-	P<5	-	(Huang et al., 2014)
**GSE42043**	Fibroblast	Skin	-	M	-	P<5	-	(Huang et al., 2014)
**GSE49053**	Fibroblast	Derm	Skin	-	-	-	FBS	(Koyanagi-Aoi et al., 2013)

F = female; M = male; P = passage; FBS = fetal bovine serum and hPL = human platelet lysate; IC = iliac crest; CF = caput femoris.

Table S2. DNAm profiles of the validation dataset (450K BeadChip)

GEO-number	Cell-Type	Source	Location	Gender	Age	Passage	Serum	References
**GSE55571**	MSC	BM	-	-	-	-	FBS	(Miyata et al., 2015)
**GSE34688**	MSC	BM	-	-	-	P<5	hPL	(Shao et al., 2013)
**GSE34688**	MSC	BM	-	-	-	P<5	hPL	(Shao et al., 2013)
**GSE34688**	MSC	BM	-	-	-	P<5	hPL	(Shao et al., 2013)
**GSE34688**	MSC	BM	-	-	-	P<5	hPL	(Shao et al., 2013)
**GSE34688**	MSC	BM	-	-	-	P<5	hPL	(Shao et al., 2013)
**GSE37067**	MSC	BM	-	-	-	P<5	hPL	(Koch et al., 2013)
**GSE37067**	MSC	BM	-	-	-	P<5	hPL	(Koch et al., 2013)
**GSE37067**	MSC	BM	-	-	-	P<5	hPL	(Koch et al., 2013)
**GSE37067**	MSC	BM	-	-	-	P<5	hPL	(Koch et al., 2013)
**GSE37067**	MSC	BM	-	-	-	P<5	hPL	(Koch et al., 2013)
**GSE37067**	MSC	BM	-	-	-	P>5	hPL	(Koch et al., 2013)
**GSE37067**	MSC	BM	-	-	-	P>5	hPL	(Koch et al., 2013)
**GSE37067**	MSC	BM	-	-	-	P>5	hPL	(Koch et al., 2013)
**GSE37067**	MSC	BM	-	-	-	P>5	hPL	(Koch et al., 2013)
**GSE52114**	MSC	BM	-	-	<40	P>5	FBS	(Fernandez et al., 2015)
**GSE52114**	MSC	BM	-	-	<40	P>5	FBS	(Fernandez et al., 2015)
**GSE52114**	MSC	BM	-	-	<40	P>5	FBS	(Fernandez et al., 2015)
**GSE52114**	MSC	BM	-	-	<40	P>5	FBS	(Fernandez et al., 2015)
**GSE52114**	MSC	BM	-	-	<40	P>5	FBS	(Fernandez et al., 2015)
**GSE52114**	MSC	BM	-	-	<40	P>5	FBS	(Fernandez et al., 2015)
**GSE52114**	MSC	BM	-	-	<40	P>5	FBS	(Fernandez et al., 2015)
**GSE52114**	MSC	BM	-	-	<40	P>5	FBS	(Fernandez et al., 2015)
**GSE52114**	MSC	BM	-	-	<40	P>5	FBS	(Fernandez et al., 2015)
**GSE52114**	MSC	BM	-	-	<40	P>5	FBS	(Fernandez et al., 2015)
**GSE52114**	MSC	BM	-	-	<40	P>5	FBS	(Fernandez et al., 2015)
**GSE52114**	MSC	BM	-	-	<40	P>5	FBS	(Fernandez et al., 2015)
**GSE52114**	MSC	BM	-	-	<40	P>5	FBS	(Fernandez et al., 2015)
**GSE52114**	MSC	BM	-	-	<40	P>5	FBS	(Fernandez et al., 2015)
**GSE52114**	MSC	BM	-	-	<40	P>5	FBS	(Fernandez et al., 2015)
**GSE52114**	MSC	BM	-	-	<40	P>5	FBS	(Fernandez et al., 2015)
**GSE52114**	MSC	BM	-	-	<40	P>5	FBS	(Fernandez et al., 2015)
**GSE52114**	MSC	BM	-	-	<40	P>5	FBS	(Fernandez et al., 2015)
**GSE52114**	MSC	BM	-	-	<40	P>5	FBS	(Fernandez et al., 2015)
**GSE52114**	MSC	BM	-	-	<40	P>5	FBS	(Fernandez et al., 2015)
**GSE52114**	MSC	BM	-	-	<40	P>5	FBS	(Fernandez et al., 2015)
**GSE52114**	MSC	BM	-	-	<40	P>5	FBS	(Fernandez et al., 2015)
**GSE52114**	MSC	BM	-	-	>40	P>5	FBS	(Fernandez et al., 2015)
**GSE52114**	MSC	BM	-	-	>40	P>5	FBS	(Fernandez et al., 2015)
**GSE52114**	MSC	BM	-	-	>40	P>5	FBS	(Fernandez et al., 2015)
**GSE52114**	MSC	BM	-	-	>40	P>5	FBS	(Fernandez et al., 2015)
**GSE52114**	MSC	BM	-	-	>40	P>5	FBS	(Fernandez et al., 2015)
**GSE52114**	MSC	BM	-	-	>40	P>5	FBS	(Fernandez et al., 2015)
**GSE52114**	MSC	BM	-	-	>40	P>5	FBS	(Fernandez et al., 2015)
**GSE52114**	MSC	BM	-	-	>40	P>5	FBS	(Fernandez et al., 2015)
**GSE52114**	MSC	BM	-	-	>40	P>5	FBS	(Fernandez et al., 2015)
**GSE52114**	MSC	BM	-	-	>40	P>5	FBS	(Fernandez et al., 2015)
**GSE52114**	MSC	BM	-	-	>40	P>5	FBS	(Fernandez et al., 2015)
**GSE52114**	MSC	BM	-	-	>40	P>5	FBS	(Fernandez et al., 2015)
**GSE55889**	MSC	AT	-	-	-	P<5	hPL	(Schellenberg et al., 2014)
**GSE55889**	MSC	AT	-	-	-	P<5	hPL	(Schellenberg et al., 2014)
**GSE55889**	MSC	AT	-	-	-	P<5	hPL	(Schellenberg et al., 2014)
**GSE55889**	MSC	AT	-	-	-	P<5	hPL	(Schellenberg et al., 2014)
**GSE55889**	MSC	AT	-	-	-	P<5	hPL	(Schellenberg et al., 2014)
**GSE55889**	MSC	AT	-	-	-	P<5	hPL	(Schellenberg et al., 2014)

**GSE55889**	MSC	AT	-	-	-	P<5	hPL	(Schellenberg et al., 2014)
**GSE55889**	MSC	AT	-	-	-	P<5	hPL	(Schellenberg et al., 2014)
**GSE57151**	MSC	AT	-	-	-	-	hPL	(Reinisch et al., 2015)
**GSE57151**	MSC	AT	-	-	-	-	hPL	(Reinisch et al., 2015)
**GSE57151**	MSC	AT	-	-	-	-	hPL	(Reinisch et al., 2015)
**GSE57151**	MSC	UC	-	-	-	-	hPL	(Reinisch et al., 2015)
**GSE57151**	MSC	UC	-	-	-	-	hPL	(Reinisch et al., 2015)
**GSE57151**	MSC	UC	-	-	-	-	hPL	(Reinisch et al., 2015)
**GSE57151**	MSC	BM	-	-	-	-	hPL	(Reinisch et al., 2015)
**GSE57151**	MSC	BM	-	-	-	-	hPL	(Reinisch et al., 2015)
**GSE57151**	MSC	BM	-	-	-	-	hPL	(Reinisch et al., 2015)
**GSE30654**	Fibroblast	Skin	-	-	-	-	FBS	(Nazor et al., 2012)
**GSE30654**	Fibroblast	Lung	-	-	-	-	FBS	(Nazor et al., 2012)
**GSE30654**	Fibroblast	Derm	-	-	-	-	FBS	(Nazor et al., 2012)
**GSE30654**	Fibroblast	Derm	-	-	-	-	FBS	(Nazor et al., 2012)
**GSE30654**	Fibroblast	Derm	-	-	-	-	FBS	(Nazor et al., 2012)
**GSE30654**	Fibroblast	Derm	-	-	-	-	FBS	(Nazor et al., 2012)
**GSE30654**	Fibroblast	Heart	-	-	-	-	FBS	(Nazor et al., 2012)
**GSE30654**	Fibroblast	Heart	-	-	-	-	FBS	(Nazor et al., 2012)
**GSE30654**	Fibroblast	Lung	-	-	-	-	FBS	(Nazor et al., 2012)
**GSE40790**	Fibroblast	Lung	-	-	-	-	FBS	(Merling et al., 2013)
**GSE40927**	Fibroblast	Skin	-	-	-	P<5	FBS	(Kurian et al., 2013)
**GSE40927**	Fibroblast	Skin	-	-	-	P<5	FBS	(Kurian et al., 2013)
**GSE40927**	Fibroblast	Skin	-	-	-	P>5	FBS	(Kurian et al., 2013)
**GSE46650**	Fibroblast	Synovial	-	-	-	-	FBS	(de la Rica et al., 2013)
**GSE46650**	Fibroblast	Synovial	-	-	-	-	FBS	(de la Rica et al., 2013)
**GSE46650**	Fibroblast	Synovial	-	-	-	-	FBS	(de la Rica et al., 2013)
**GSE46650**	Fibroblast	Synovial	-	-	-	-	FBS	(de la Rica et al., 2013)
**GSE46650**	Fibroblast	Synovial	-	-	-	-	FBS	(de la Rica et al., 2013)
**GSE46650**	Fibroblast	Synovial	-	-	-	-	FBS	(de la Rica et al., 2013)
**GSE46650**	Fibroblast	Synovial	-	-	-	-	FBS	(de la Rica et al., 2013)
**GSE46650**	Fibroblast	Synovial	-	-	-	-	FBS	(de la Rica et al., 2013)
**GSE46650**	Fibroblast	Synovial	-	-	-	-	FBS	(de la Rica et al., 2013)
**GSE46650**	Fibroblast	Synovial	-	-	-	-	FBS	(de la Rica et al., 2013)
**GSE46650**	Fibroblast	Synovial	-	-	-	-	FBS	(de la Rica et al., 2013)
**GSE46650**	Fibroblast	Synovial	-	-	-	-	FBS	(de la Rica et al., 2013)
**GSE53096**	Fibroblast	Derm	-	-	-	-	FBS	(Ma et al., 2014)
**GSE53918**	Fibroblast	Cornea	-	-	-	-	FBS	(Sareen et al., 2014)
**GSE53918**	Fibroblast	Cornea	-	-	-	-	FBS	(Sareen et al., 2014)
**GSE53918**	Fibroblast	Cornea	-	-	-	-	FBS	(Sareen et al., 2014)
**GSE54115**	Fibroblast	Skin	-	-	-	P>5	FBS	-
**GSE54115**	Fibroblast	Skin	-	-	-	P>5	FBS	-
**GSE54848**	Fibroblast	Derm	-	-	-	-	FBS	(Ohnuki et al., 2014)
**GSE54848**	Fibroblast	Derm	-	-	-	-	FBS	(Ohnuki et al., 2014)
**GSE54848**	Fibroblast	Derm	-	-	-	-	FBS	(Ohnuki et al., 2014)
**GSE57151**	Fibroblast	-	-	-	-	-	FBS	(Reinisch et al., 2015)
**GSE57151**	Fibroblast	-	-	-	-	-	FBS	(Reinisch et al., 2015)
**GSE57151**	Fibroblast	-	-	-	-	-	FBS	(Reinisch et al., 2015)
**GSE57992**	Fibroblast	Aminiotic	-	-	-	-	FBS	(He et al., 2014)
**GSE57992**	Fibroblast	-	-	-	-	-	FBS	(He et al., 2014)
**GSE61461**	Fibroblast	Skin	-	-	-	-	FBS	(Johannesson et al., 2014)
**GSE61461**	Fibroblast	Skin	-	-	-	-	FBS	(Johannesson et al., 2014)

P = passage; FBS = fetal bovine serum; hPL = human platelet lysate; IC = iliac crest; CF = caput femoris; UC = umbilical cord.

Table S3. Primary cell preparations used for pyrosequencing analysis

Sample ID	Tissue Source	Age	Gender	Passage
MSC 1	Bone Marrow	69	Female	3
MSC 2	Bone Marrow	84	Male	2
MSC 3	Bone Marrow	50	Female	1
MSC 4	Bone Marrow	33	Female	2
MSC 5	Bone Marrow	59	Male	1
MSC 6	Bone Marrow	50	Male	2
MSC 7	Bone Marrow	30	Male	2
MSC 8	Bone Marrow	70	Female	2
MSC 9	Bone Marrow	87	Male	1
MSC 10	Bone Marrow	73	Male	1
MSC 11	Bone Marrow	66	Male	1
MSC 12	Bone Marrow	67	Male	2
MSC 13	Adipose Tissue	46	Male	4
MSC 14	Adipose Tissue	43	Female	2
MSC 15	Adipose Tissue	48	Female	2
MSC 16	Adipose Tissue	50	Female	3
MSC 17	Adipose Tissue	43	Female	1
MSC 18	Adipose Tissue	19	Female	3
MSC 19	Adipose Tissue	24	Male	1
MSC 20	Adipose Tissue	23	Male	1
MSC 21	Adipose Tissue	24	Female	1
MSC 22	Adipose Tissue	29	Female	2
Fibroblast 1	Dermis (Breast)	42	Female	2
Fibroblast 2	Dermis (Abdomen)	62	Female	2
Fibroblast 3	Dermis (Breast)	43	Female	2
Fibroblast 4	Dermis (Breast)	18	Female	2
Fibroblast 5	Dermis (Arm)	63	Female	2
Fibroblast 6	Dermis (Abdomen)	73	Female	2
Fibroblast 7	Dermis (Ear)	64	Female	2
Fibroblast 8	Dermis (Breast)	60	Female	3
Fibroblast 9	Dermis (Abdomen)	23	Female	11
Fibroblast 10	Dermis	60	Female	10
Fibroblast 11	Dermis	60	Female	5
Fibroblast 12	Dermis (Breast)	60	Female	16

Table S4. Primers for pyrosequencing assays

Primer	CpG ID	Sequence
C3orf35_F Biotin	cg22286764	5`-TGTGTGTATTTTGTTGTTTATTTTTTGGGTTTAGGAGAA-3`
C3orf35_R		5`-CCTCCCTTAAAATCAATCTCCAATCATTTAATTAACTT-3`
C3orf35_seq		5`-AACTTAACTACAATCATTCACA-3`
CIDEC_F	cg05684195	5`-TGAGTAGATAATTTAATTTAGGGTTGAAGAAGTTTTGT-3`
CIDEC _R Biotin		5`-CATCCCCAAAAATATAAAATAATATAACTCACCTCC-3`
CIDEC_seq		5`-AGATTTGTTTTTGTTTATGG-3`
SLC41A2_F	cg27149093	5`-TGTGTTTGTTTTTTTGGTTTTTTTGGTAAATTA-3`
SLC41A2_R Biotin		5`-CCAAATCCAATCCTTCTATAAAACTTCTAAACATCTT-3`
SLC41A2_seq		5`-GGGAATTATAGTAGATGAATTAG-3`
TM4SF1_F Biotin	cg08124030	5`-ATAAAGAGAAGGGGGAGAAAATTTAGTAGATTATTATGTG-3`
TM4SF1_R		5`-TTTCCCCATTAAAAAAATAAAACAAAATATTAACC-3`
TM4SF1_seq		5`-AAAAATAAAACAAAATATTAACC-3`

_F = forward primer; _R = reverse primer.

Supplemental Experimental Procedures

Bioinformatic procedures

The DNAm datasets (Supplemental Tables S1 and S2) were carefully evaluated with regard to the corresponding publications. CpGs linked to X and Y chromosomes were excluded and we focused on 25,014 CpGs represented by 27K and 450K BeadChips. DNAm levels (β-values) were quantile normalized using the R package *lumi*. Principal component analysis (PCA) was performed with the R package *stats* using singular value decomposition of the data matrix. Significant differentially methylated CpG-sites were identified by Limma adjusted t-test (R package *limma*; $p < 0.05$).

Permutation assays

Bootstrapping was performed on the 27k BeadChip training set to estimate likelihood that the CpGs of the Epi-MSC-Score provide reproducible results according to (i) difference in mean DNAm in MSCs *versus* fibroblasts, and (ii) variation in DNAm levels within each of the two cell types. This method was performed 1000 times. The results revealed that the CpG site in *CIDEC* (cg05684195) was on rank four (88% of replicates) and the CpG site in *C3orf35* (cg22286764) was on rank eight (73% of replicates) of all 25,014 CpGs. In analogy, we performed the same experiment for the DNAm changes between MSCs from BM *versus* AT: *TM4SF1* (cg08124030) was on rank one (100% of the replicates) and *SLC41A2* (cg27149093) was on rank nine (93% of the replicates). This reanalysis supported the notion that the CpGs of the Epi-MSC-Score and of the Epi-Tissue-Score are within the most stable CpGs sites for these comparisons.

Analysis of Epi-MSC-Score in other cell types

DNAm profiles (450k) of MSCs that were subsequently used for reprogramming into iPSCs (GSE34688), of iPSCs (GSE34688), and of iPS-MSCs (GSE54767) were retrieved from GEO. Furthermore, we applied the Epi-MSC-Score to DNAm profiles of blood (GSE39981), monocytes (GSE59339), and macrophages (GSE31680). Overall, Epi-MSC-Score of these hematopoietic cells was close to zero, indicating that potentially contaminating macrophages would hardly impact on the Epi-MSC-Score – in this regard the Epi-MSC-Score is no substitute for immunophenotypic analysis.

Analysis of differential gene expression in genes of Epi-MSC-Score and Epi-Tissue-Score

To estimate if DNAm changes might be reflected in differential gene expression we downloaded microarray data from GEO (all Illumina HumanHT-12 v4 platform; GPL10558). For analysis of gene expression of *C3orf35* and *CIDEC* (associated with Epi-MSC-Score) in MSCs *versus* fibroblasts we used the following profiles: for MSCs: GSM1050328, GSM1050329, GSM1050330, GSM1050331, GSM1050332, GSM1050333, GSM1128574, GSM1128575, GSM1276944, GSM1276947, GSM1276948, GSM1276949, GSM1350082, GSM1350083, GSM1350088, GSM1350089, GSM1350090, GSM1515746, GSM1515747, GSM1515748, GSM1515749, GSM1515750, GSM1515751, GSM1515752; and for fibroblasts: GSM786856, GSM786857, GSM1348171, GSM1348172, GSM1348173, GSM860982, GSM860983, GSM860984, GSM1359297, GSM1359298, GSM1359309, GSM1359310, GSM1381443, GSM1586080, GSM1586082, GSM1586085, GSM1586089, GSM1329667, GSM1329668, GSM1664886, GSM1664890, GSM1664894. To estimate gene expression levels of *SLC41A2* and *TM4SF1* (associated with CpGs of the Epi-Tissue-Score) we utilized the following profiles for MSCs from bone marrow: GSM1050328, GSM1050329, GSM1050330, GSM1050331, GSM1050332, GSM1050333, GSM1128574, GSM1128575, GSM1276944, GSM1276947, GSM1276948, GSM1276949, GSM1350082, GSM1350083, GSM1350088, GSM1350089, GSM1350090; and for MSCs from adipose tissue: GSM1515746, GSM1515747, GSM1515748, GSM1515749, GSM1515750, GSM1515751, GSM1515752. Data were quantile normalized for comparison of relative gene expression levels.

Additional information on clonal analysis of MSCs

After two weeks, individual clones with confluence of 50% or more were counted to estimate the *CFU-Fs* (colony-forming unit fibroblast-like) frequency with the *L-Calc Software* (Stem Cell Technologies, Canada) (Schellenberg et al., 2012) and then harvested. In addition, we used independent 96-well plates, that were either differentiated towards osteogenic or adipogenic lineages for two additional weeks and stained with Alizarin Red or BODIPY, respectively (Schellenberg et al., 2012). After absorbance measurement (Tecan PRO, Switzerland) and fluorescence analysis (EVOS, Life Technologies, USA) the DNA was harvested for pyrosequencing.

Supplemental References:

Berdasco, M., Melguizo, C., Prados, J., Gomez, A., Alaminos, M., Pujana, M.A., Lopez, M., Setien, F., Ortiz, R., Zafra, I., *et al.* (2012). DNA methylation plasticity of human adipose-derived stem cells in lineage commitment. Am J Pathol *181*, 2079-2093.

Bork, S., Pfister, S., Witt, H., Horn, P., Korn, B., Ho, A.D., and Wagner, W. (2010). DNA methylation pattern changes upon long-term culture and aging of human mesenchymal stromal cells. Aging cell *9*, 54-63.

de la Rica, L., Urquiza, J.M., Gomez-Cabrero, D., Islam, A.B., Lopez-Bigas, N., Tegner, J., Toes, R.E., and Ballestar, E. (2013). Identification of novel markers in rheumatoid arthritis through integrated analysis of DNA methylation and microRNA expression. J Autoimmun *41*, 6-16.

Fernandez, A.F., Bayon, G.F., Urdinguio, R.G., Torano, E.G., Garcia, M.G., Carella, A., Petrus-Reurer, S., Ferrero, C., Martinez-Camblor, P., Cubillo, I., *et al.* (2015). H3K4me1 marks DNA regions hypomethylated during aging in human stem and differentiated cells. Genome Res *25*, 27-40.

He, W., Kang, X., Du, H., Song, B., Lu, Z., Huang, Y., Wang, D., Sun, X., Yu, Y., and Fan, Y. (2014). Defining differentially methylated regions specific for the acquisition of pluripotency and maintenance in human pluripotent stem cells via microarray. PloS one *9*, e108350.

Huang, K., Shen, Y., Xue, Z., Bibikova, M., April, C., Liu, Z., Cheng, L., Nagy, A., Pellegrini, M., Fan, J.B., *et al.* (2014). A panel of CpG methylation sites distinguishes human embryonic stem cells and induced pluripotent stem cells. Stem Cell Reports *2*, 36-43.

Johannesson, B., Sagi, I., Gore, A., Paull, D., Yamada, M., Golan-Lev, T., Li, Z., LeDuc, C., Shen, Y., Stern, S., *et al.* (2014). Comparable frequencies of coding mutations and loss of imprinting in human pluripotent cells derived by nuclear transfer and defined factors. Cell Stem Cell *15*, 634-642.

Koch, C.M., Joussen, S., Schellenberg, A., Lin, Q., Zenke, M., and Wagner, W. (2012). Monitoring of cellular senescence by DNA-methylation at specific CpG-sites. Aging cell *11*, 366-369.

Koch, C.M., Reck, K., Shao, K., Lin, Q., Joussen, S., Ziegler, P., Walenda, G., Drescher, W., Opalka, B., May, T., *et al.* (2013). Pluripotent stem cells escape from senescence-associated DNA methylation changes. Genome Res *23*, 248-259.

Koch, C.M., Suschek, C.V., Lin, Q., Bork, S., Goergens, M., Joussen, S., Pallua, N., Ho, A.D., Zenke, M., and Wagner, W. (2011). Specific age-associated DNA methylation changes in human dermal fibroblasts. PloS one *6*, e16679.

Koyanagi-Aoi, M., Ohnuki, M., Takahashi, K., Okita, K., Noma, H., Sawamura, Y., Teramoto, I., Narita, M., Sato, Y., Ichisaka, T., *et al.* (2013). Differentiation-defective phenotypes revealed by large-scale analyses of human pluripotent stem cells. Proc Natl Acad Sci U S A *110*, 20569-20574.

Kurian, L., Sancho-Martinez, I., Nivet, E., Aguirre, A., Moon, K., Pendaries, C., Volle-Challier, C., Bono, F., Herbert, J.M., Pulecio, J., *et al.* (2013). Conversion of human fibroblasts to angioblast-like progenitor cells. Nat Methods *10*, 77-83.

Ma, H., Morey, R., O'Neil, R.C., He, Y., Daughtry, B., Schultz, M.D., Hariharan, M., Nery, J.R., Castanon, R., Sabatini, K., *et al.* (2014). Abnormalities in human pluripotent cells due to reprogramming mechanisms. Nature *511*, 177-183.

Merling, R.K., Sweeney, C.L., Choi, U., De Ravin, S.S., Myers, T.G., Otaizo-Carrasquero, F., Pan, J., Linton, G., Chen, L., Koontz, S., *et al.* (2013). Transgene-free iPSCs generated from small volume peripheral blood nonmobilized CD34+ cells. Blood *121*, e98-107.

Miyata, K., Miyata, T., Nakabayashi, K., Okamura, K., Naito, M., Kawai, T., Takada, S., Kato, K., Miyamoto, S., Hata, K., *et al.* (2015). DNA methylation analysis of human myoblasts during in vitro myogenic differentiation: de novo methylation of promoters of muscle-related genes and its involvement in transcriptional down-regulation. Hum Mol Genet *24*, 410-423.

Navab, R., Strumpf, D., Bandarchi, B., Zhu, C.Q., Pintilie, M., Ramnarine, V.R., Ibrahimov, E., Radulovich, N., Leung, L., Barczyk, M., *et al.* (2011). Prognostic gene-expression signature of carcinoma-associated fibroblasts in non-small cell lung cancer. Proc Natl Acad Sci U S A *108*, 7160-7165.

Nazor, K.L., Altun, G., Lynch, C., Tran, H., Harness, J.V., Slavin, I., Garitaonandia, I., Muller, F.J., Wang, Y.C., Boscolo, F.S., *et al.* (2012). Recurrent variations in DNA methylation in human pluripotent stem cells and their differentiated derivatives. Cell Stem Cell *10*, 620-634.

Nishino, K., Toyoda, M., Yamazaki-Inoue, M., Fukawatase, Y., Chikazawa, E., Sakaguchi, H., Akutsu, H., and Umezawa, A. (2011). DNA methylation dynamics in human induced pluripotent stem cells over time. PLoS Genet *7*, e1002085.

Ohm, J.E., Mali, P., Van Neste, L., Berman, D.M., Liang, L., Pandiyan, K., Briggs, K.J., Zhang, W., Argani, P., Simons, B., *et al.* (2010). Cancer-related epigenome changes associated with reprogramming to induced pluripotent stem cells. Cancer Res *70*, 7662-7673.

Ohnuki, M., Tanabe, K., Sutou, K., Teramoto, I., Sawamura, Y., Narita, M., Nakamura, M., Tokunaga, Y., Nakamura, M., Watanabe, A., *et al.* (2014). Dynamic regulation of human endogenous retroviruses mediates factor-induced reprogramming and differentiation potential. Proc Natl Acad Sci U S A *111*, 12426-12431.

Reinisch, A., Etchart, N., Thomas, D., Hofmann, N.A., Fruehwirth, M., Sinha, S., Chan, C.K., Senarath-Yapa, K., Seo, E.Y., Wearda, T., *et al.* (2015). Epigenetic and in vivo comparison of diverse MSC sources reveals an endochondral signature for human hematopoietic niche formation. Blood *125*, 249-260.

Sareen, D., Saghizadeh, M., Ornelas, L., Winkler, M.A., Narwani, K., Sahabian, A., Funari, V.A., Tang, J., Spurka, L., Punj, V., *et al.* (2014). Differentiation of human limbal-derived induced pluripotent stem cells into limbal-like epithelium. Stem Cells Transl Med *3*, 1002-1012.

Schellenberg, A., Joussen, S., Moser, K., Hampe, N., Hersch, N., Hemeda, H., Schnitker, J., Denecke, B., Lin, Q., Pallua, N., *et al.* (2014). Matrix elasticity, replicative senescence and DNA methylation patterns of mesenchymal stem cells. Biomaterials *35*, 6351-6358.

Schellenberg, A., Lin, Q., Schuler, H., Koch, C.M., Joussen, S., Denecke, B., Walenda, G., Pallua, N., Suschek, C.V., Zenke, M., *et al.* (2011). Replicative senescence of mesenchymal stem cells causes DNA-methylation changes which correlate with repressive histone marks. Aging (Albany NY) *3*, 873-888.

Schellenberg, A., Stiehl, T., Horn, P., Joussen, S., Pallua, N., Ho, A.D., and Wagner, W. (2012). Population dynamics of mesenchymal stromal cells during culture expansion. Cytotherapy *14*, 401-411.

Sempere, J.M., Martinez-Peinado, P., Arribas, M.I., Reig, J.A., De La Sen, M.L., Zubcoff, J.J., Fraga, M.F., Fernandez, A.F., Santana, A., and Roche, E. (2014). Single cell-derived clones from human adipose stem cells present different immunomodulatory properties. Clin Exp Immunol *176*, 255-265.

Shao, K., Koch, C., Gupta, M.K., Lin, Q., Lenz, M., Laufs, S., Denecke, B., Schmidt, M., Linke, M., Hennies, H.C., *et al.* (2013). Induced pluripotent mesenchymal stromal cell clones retain donor-derived differences in DNA methylation profiles. Mol Ther *21*, 240-250.
